# Supplementary material for: Enhanced AAV transduction across preclinical CNS models: A comparative study in human brain organoids with cross-species evaluations
Source: Mol Ther Nucleic Acids. 2024 Jun 28;35(3):102264. doi: 10.1016/j.omtn.2024.102264 (PMC11301180; doi:10.1016/j.omtn.2024.102264)
Supplement: Document S1. Figures S1‒S4 and Table S1 [file mmc1.pdf]

OMTN, Volume *35*

Supplemental information

Enhanced AAV transduction across preclinical CNS models: A comparative study in human brain organoids with cross-species evaluations

Matthieu Drouyer, Jessica Merjane, Teodora Nedelkoska, Adrian Westhaus, Suzanne Scott, Scott Lee, Peter G.R. Burke, Simon McMullan, Jose L. Lanciego, Ana F. Vicente, Ricardo Bugallo, Carmen Unzu, Gloria González-Aseguinolaza, Anai Gonzalez-Cordero, and Leszek Lisowski

Supplemental Figures

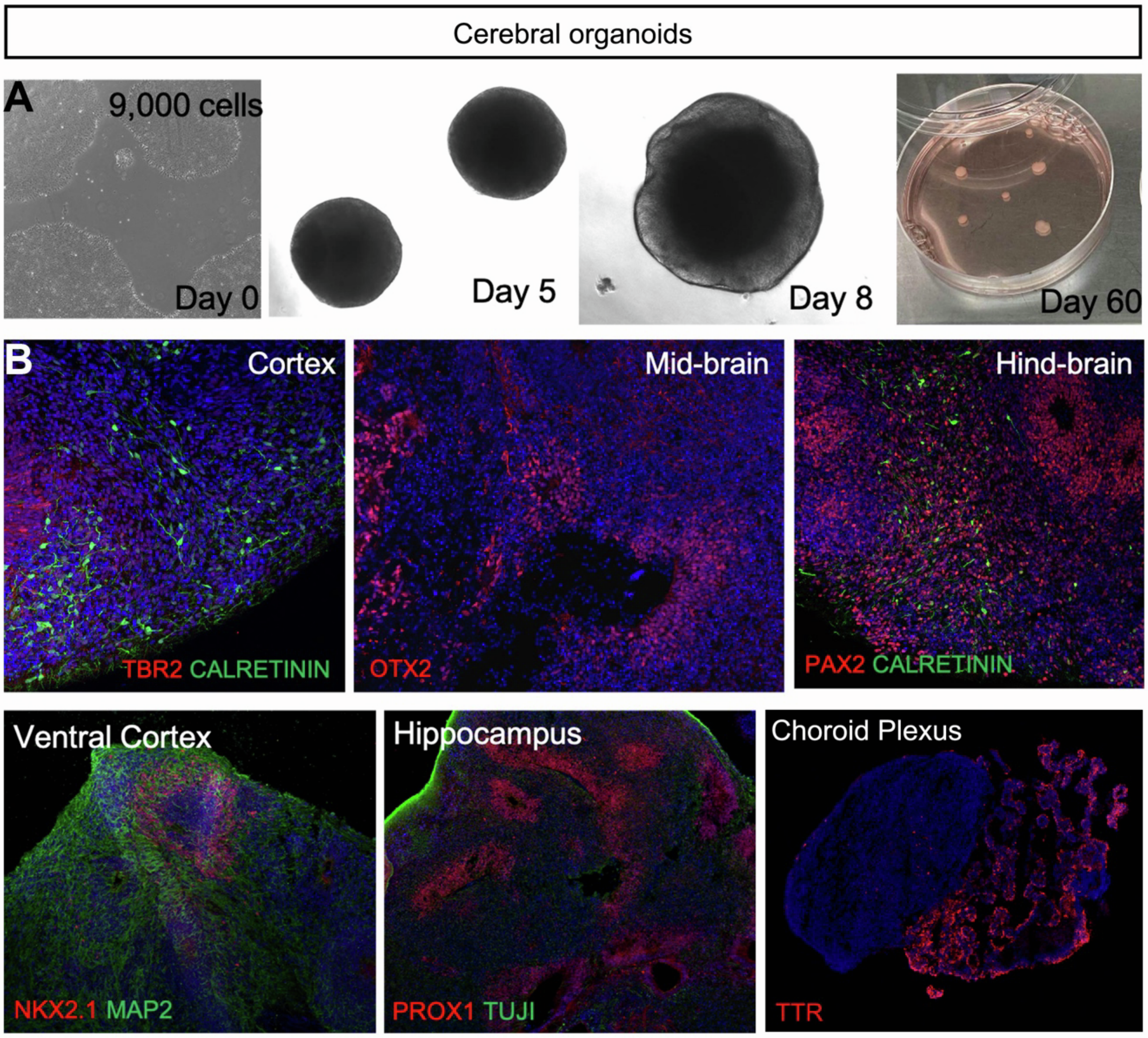

Figure S1. Characterization of human cerebral organoids. (**A**) Bright-field images of organoids induced from human iPSCs. (**B**) Cerebral organoids recapitulate various brain region identities. Staining for various brain region identities: cortex, Tbr2; mid-brain, Otx2; hind-brain, Pax2, ventral cortex, Nkx2.1; hippocampus, Prox1 and choroid plexus, TTR. Immunohistochemistry for neuronal cell marker (Tuj1, MAP2 and Calretinin). Nuclei were visualized with DAPI (blue).

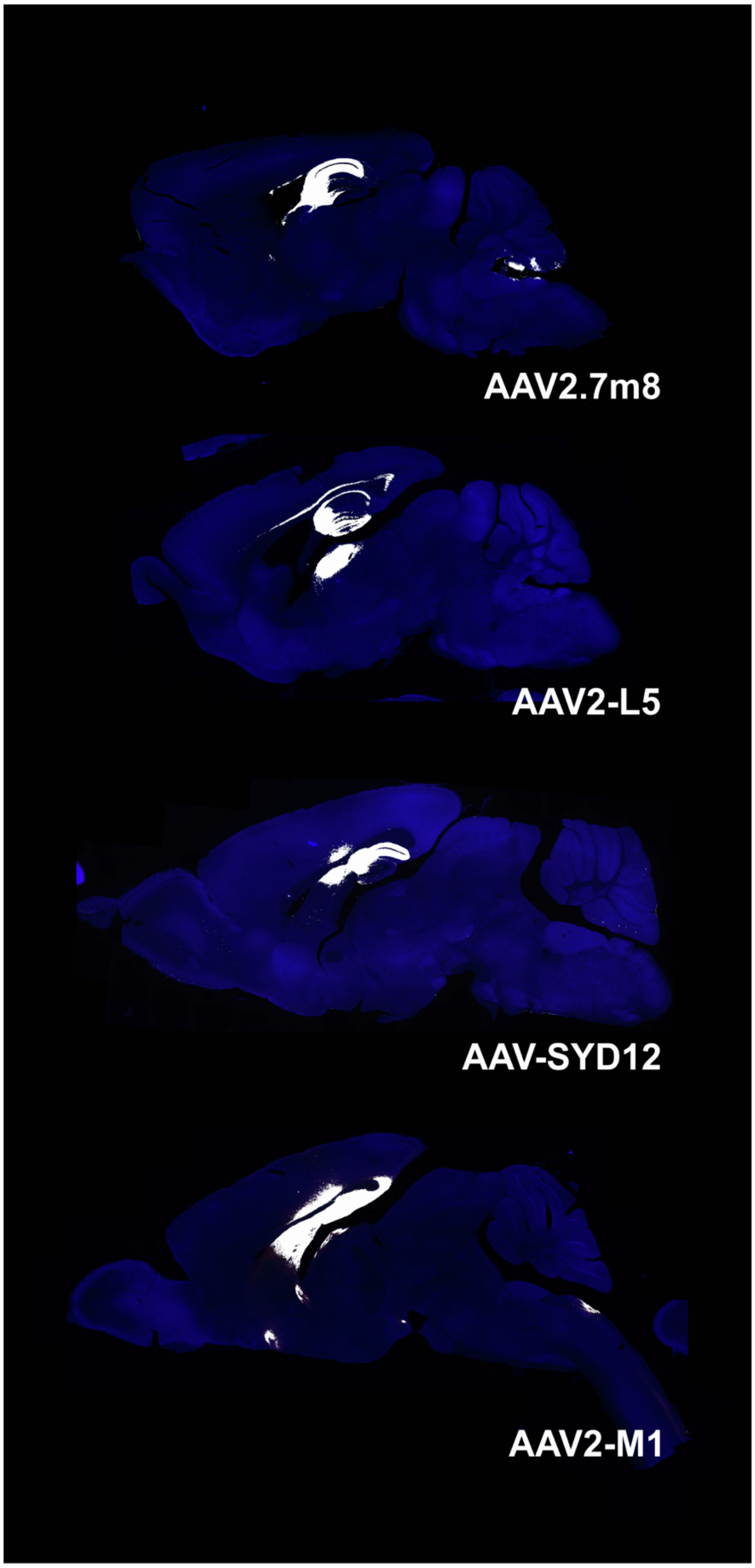

Figure S2. Threshold masks used for quantification of vector efficacy. An ImageJ macro was used to identify pixels that exceeded a threshold fluorescence intensity; the area of each image contained by the mask, and the average intensity of pixels within, were compared between vectors. Masks corresponding to images shown in Figure 5 are shown for comparison.

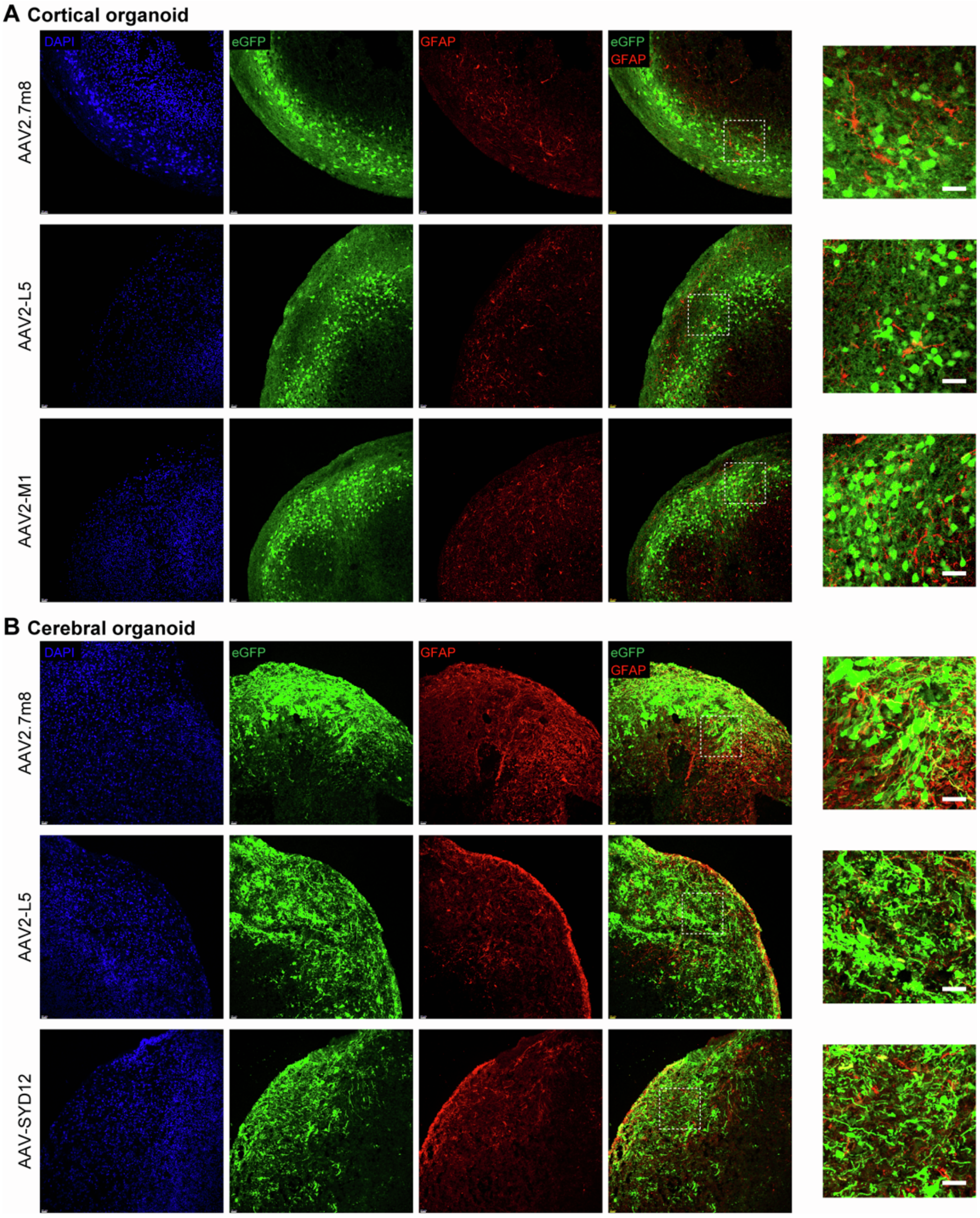

Figure S3. Immunofluorescence analysis of GFAP in cortical and cerebral organoids. (**A**) (left) eGFP reporter expression in cortical organoids following transduction with AAV2.7m8, AAV2-L5 and AAV2-M1 variants. Staining with GFAP was used to identify glia cells (n = 1 organoid, dose: 1 × 10^{10} vg per organoid, 2 weeks of expression). (right) High magnification images of selected region. Blue: DAPI (nuclei); green: vector-encoded eGFP; red: astrocytes and an overlay of green and red. Scale bars, 20µm. (**B**) (left) eGFP reporter expression in cerebral organoids following transduction with AAV2.7m8, AAV2-L5 and AAV-SYD12 variants. Staining with GFAP was used to identify glia cells (n = 1 organoid, dose: 1 × 10^{10} vg per organoid, 2 weeks of expression). (right) High magnification images of selected region. Blue: DAPI (nuclei); green: vector-encoded eGFP; red: astrocytes and an overlay of green and red. Scale bars, 20µm.

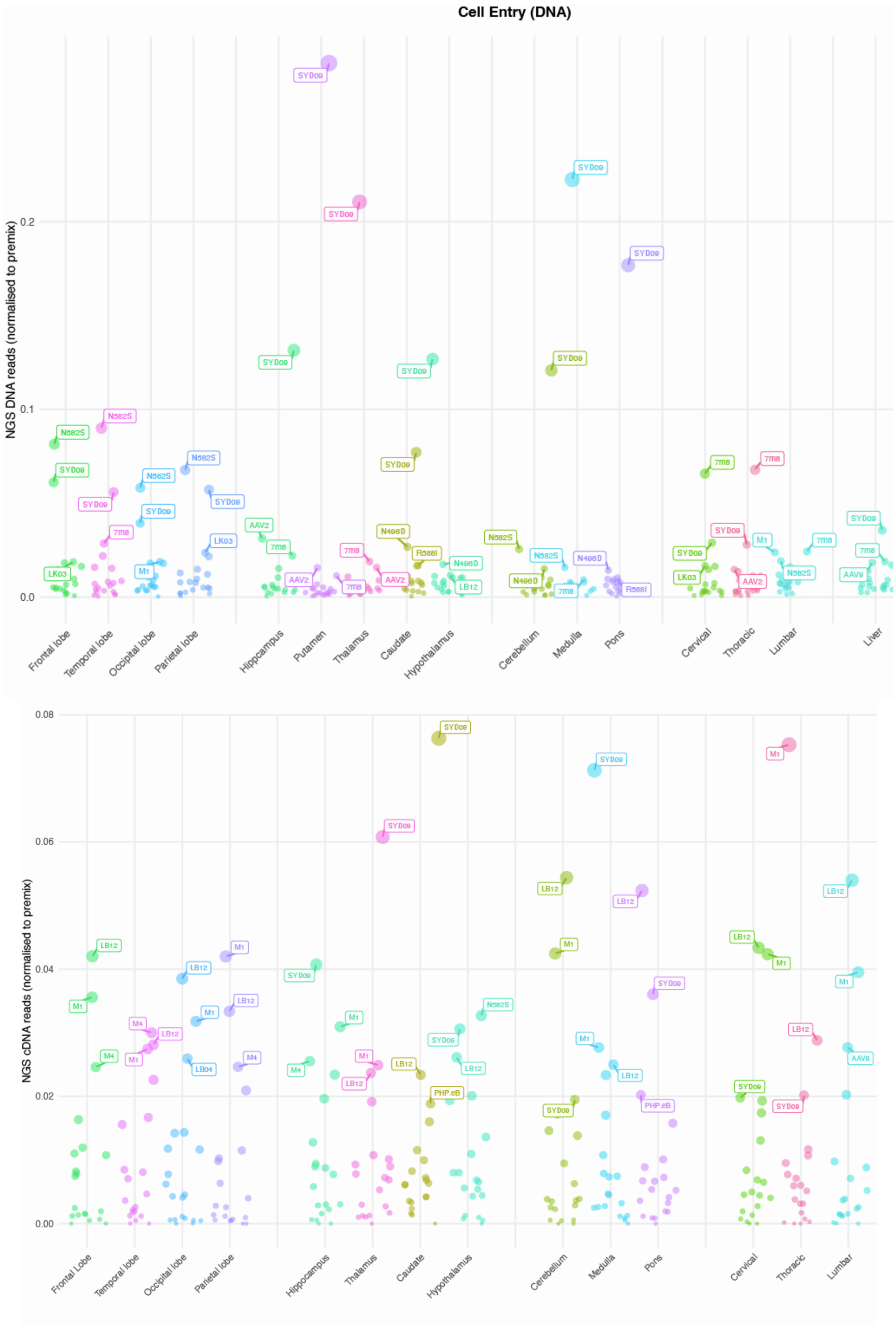

Figure S3. AAV biodistribution in CNS areas and liver in non-human primate following Intra-cisterna magna injection. NGS analysis of the transduction efficiency of each AAV variant at cell entry (DNA reads) and functional expression (RNA/cDNA reads) for each region within the CNS and liver. Bubble plots depict the percentage of NGS reads for each variant. Each bubble represents one AAV variant. Bubble size is proportional to percentage number. Top three AAV serotypes for each tissue is indicated.

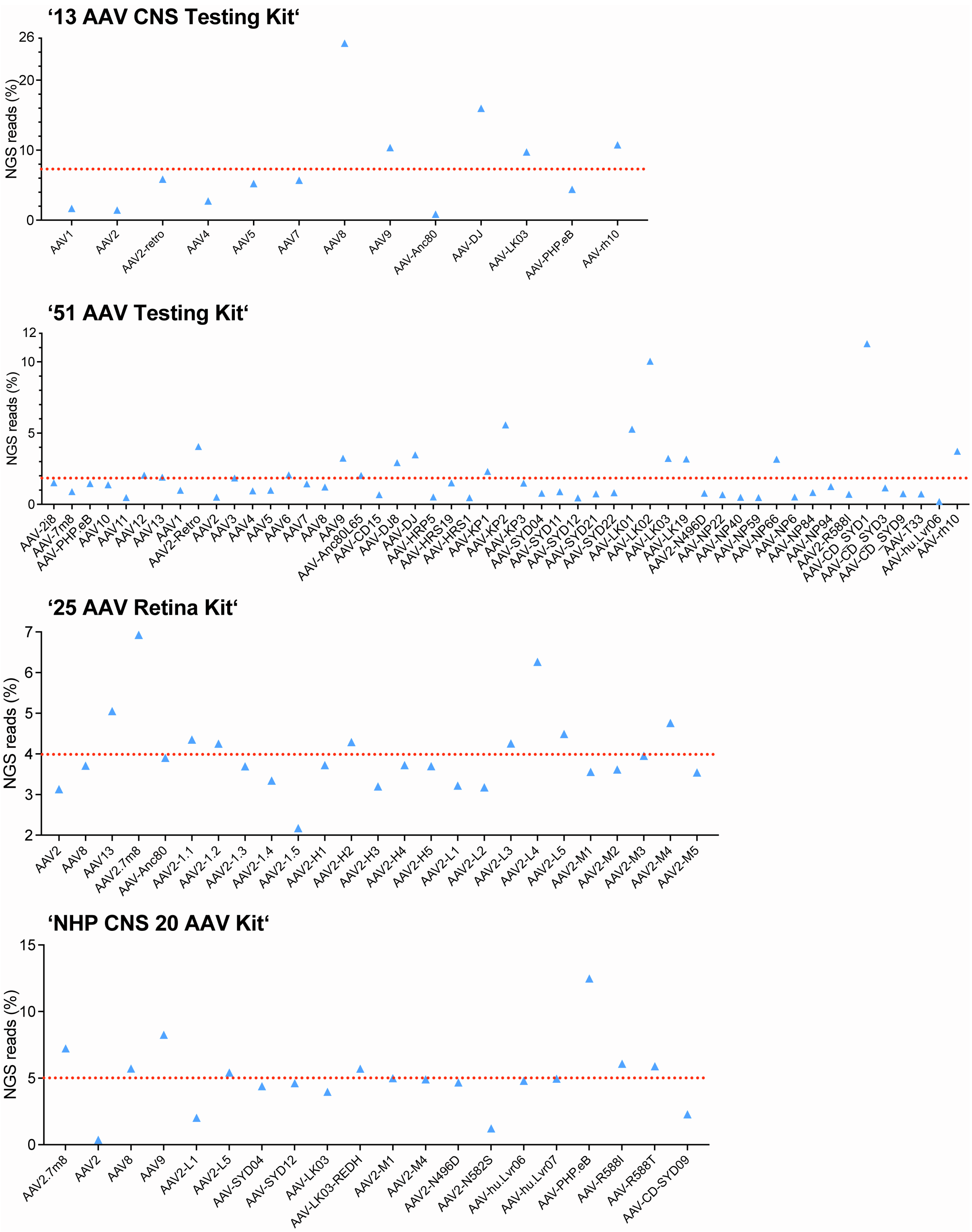

Figure S4. Distribution of each capsid variant in the 4 different ‘AAV Testing kits’ used in this study. The expected percentage contribution of individual variants is indicated by the red dotted line.

Table S1. AAV capsid variants used in the study.

AAV Variant	Capsid Origin	AAV CNS Testing	51 AAV Testing kit	25 AAV Retina kit	NHP CNS 20 AAV kit	Reference
AAV1	Natural serotype	X	X			PMID: 14325163
AAV2	Natural serotype	X	X	X	X	PMID: 5227666
AAV3b	Natural serotype		X			PMID: 5227666
AAV4	Natural serotype	X	X			PMID: 9261407
AAV5	Natural serotype	X	X			PMID: 6324476
AAV6	Natural serotype		X			PMID: 5227666
AAV7	Natural serotype	X	X			PMID: 12192090
AAV8	Natural serotype	X	X	X	X	PMID: 12192090
AAV9	Natural serotype	X	X		X	PMID: 15163731
AAV10	Natural serotype		X			PMID: 15567432
AAV11	Natural serotype		X			PMID: 15567432
AAV12	Natural serotype		X			PMID: 18045941
AAV13	Natural serotype		X	X		PMID: 18524816
AAV-rh10	Natural isolate	X	X			PMID: 12716974
AAV-DJ	DNA family shuffling	X	X			PMID: 18400866
AAV-DJ8	DNA family shuffling		X			PMID: 18400866
AAV-LK01	DNA family shuffling		X			PMID: 24390344
AAV-LK02	DNA family shuffling		X			PMID: 24390344
AAV-LK03	DNA family shuffling	X	X		X	PMID: 24390344
AAV-LK19	DNA family shuffling		X			PMID: 24390344
AAV-NP22	DNA family shuffling		X			PMID: 30101152
AAV-NP40	DNA family shuffling		X			PMID: 29055620
AAV-NP59	DNA family shuffling		X			PMID: 29055620
AAV-NP6	DNA family shuffling		X			PMID: 30101152
AAV-NP66	DNA family shuffling		X			PMID: 30101152
AAV-NP84	DNA family shuffling		X			PMID: 29055620
AAV-NP94	DNA family shuffling		X			PMID: 30101152
AAV-7m8	Peptide display on AAV2		X	X	X	PMID: 23761039
AAV-Anc80L65	Reconstructed capsid	X	X	X		PMID: 26235624
AAV-PHP.eB	Peptide display on AAV9	X	X		X	PMID: 28671695
AAV-2i8	Domain swapping		X			PMID: 20037580
AAV2-retro	Peptide display on AAV2	X	X			PMID: 27720486
AAV-CD15	DNA family shuffling		X			Our lab
AAV-HRP5	DNA family shuffling		X			Our lab
AAV-HRS19	DNA family shuffling		X			Our lab
AAV-HRS1	DNA family shuffling		X			Our lab
AAV-KP1	DNA family shuffling		X			PMID: 31723052
AAV-KP2	DNA family shuffling		X			PMID: 31723052
AAV-KP3	DNA family shuffling		X			PMID: 31723052
AAV-SYD04	DNA family shuffling		X		X	Our lab
AAV-SYD11	DNA family shuffling		X			Our lab
AAV-SYD12	DNA family shuffling		X		X	Our lab
AAV2-RC01	Peptide display on AAV2		X			Our lab
AAV2-RC02	Peptide display on AAV2		X			Our lab
AAV2-N496D	Rational Design		X		X	Our lab
AAV2-R588I	Rational Design		X		X	Our lab
AAV-CD-SYD01	DNA family shuffling		X			Our lab
AAV-CD-SYD03	DNA family shuffling		X			Our lab
AAV-CD-SYD09	DNA family shuffling		X		X	Our lab
AAV-T33	DNA family shuffling		X			Our lab
AAV-hu.Lvr06	Natural isolate		X		X	Our lab
AAV2-N582S	Rational Design				X	Our lab
AAV2-R588T	Rational Design				X	Our lab
AAV-LK03-REDH	Rational Design				X	Our lab
AAV-hu.Lvr07	Natural isolate				X	Our lab
AAV2-1.1	Peptide display on AAV2			X		Our lab
AAV2-1.2	Peptide display on AAV2			X		Our lab
AAV2-1.3	Peptide display on AAV2			X		Our lab
AAV2-1.4	Peptide display on AAV2			X		Our lab
AAV2-1.5	Peptide display on AAV2			X		Our lab
AAV2-H1	Peptide display on AAV2			X		Our lab
AAV2-H2	Peptide display on AAV2			X		Our lab
AAV2-H3	Peptide display on AAV2			X		Our lab
AAV2-H4	Peptide display on AAV2			X		Our lab
AAV2-H5	Peptide display on AAV2			X		Our lab
AAV2-L1	Peptide display on AAV2			X	X	Our lab
AAV2-L2	Peptide display on AAV2			X		Our lab
AAV2-L3	Peptide display on AAV2			X		Our lab
AAV2-L4	Peptide display on AAV2			X		Our lab
AAV2-L5	Peptide display on AAV2			X	X	Our lab
AAV2-M1	Peptide display on AAV2			X	X	Our lab
AAV2-M2	Peptide display on AAV2			X		Our lab
AAV2-M3	Peptide display on AAV2			X		Our lab
AAV2-M4	Peptide display on AAV2			X	X	Our lab
AAV2-M5	Peptide display on AAV2			X		Our lab
