# Supplementary material for: Enhanced AAV transduction across preclinical CNS models: A comparative study in human brain organoids with cross-species evaluations
Source: Mol Ther Nucleic Acids. 2024 Jun 28;35(3):102264. doi: 10.1016/j.omtn.2024.102264 (PMC11301180; doi:10.1016/j.omtn.2024.102264)
Supplement: Document S2. Article plus supplemental information [file mmc2.pdf]

Molecular Therapy
Nucleic Acids
Original Article

Enhanced AAV transduction across preclinical CNS models: A comparative study in human brain organoids with cross-species evaluations

Matthieu Drouyer,[1,10] Jessica Merjane,[1] Teodora Nedelkoska,[2] Adrian Westhaus,[1,8] Suzanne Scott,[1,3] Scott Lee,[4] Peter G.R. Burke,[2] Simon McMullan,[2] Jose L. Lanciego,[5] Ana F. Vicente,[5,9] Ricardo Bugallo,[5] Carmen Unzu,[5] Gloria González-Aseguinolaza,[5] Anai Gonzalez-Cordero,[4] and Leszek Lisowski[1,6,7]

[1]Translational Vectorology Research Unit, Children's Medical Research Institute, Faculty of Medicine and Health, The University of Sydney, Westmead, NSW 2145, Australia; [2]Macquarie Medical School, Faculty of Medicine, Health & Human Sciences, Macquarie University, Sydney, NSW 2109, Australia; [3]Australian e-Health Research Centre, Commonwealth Scientific and Industrial Research Organisation, Sydney, NSW 2145, Australia; [4]Stem Cell Medicine and Stem Cell and Organoid Facility, Children's Medical Research Institute, Faculty of Medicine and Health, The University of Sydney, Westmead, NSW 2145, Australia; [5]DNA & RNA Medicine Division, IdiSNA, Instituto de Investigación Sanitaria de Navarra, Universidad de Navarra, CIMA, Pamplona, Spain; [6]Australian Genome Therapeutics Centre, Children's Medical Research Institute and Sydney Children's Hospitals Network, Westmead, NSW 2145, Australia; [7]Laboratory of Molecular Oncology and Innovative Therapies, Military Institute of Medicine - National Research Institute, 04-349 Warsaw, Poland

Viral vectors based on recombinant adeno-associated virus (rAAV) have become the most widely used system for therapeutic gene delivery in the central nervous system (CNS). Despite clinical safety and efficacy in neurological applications, a barrier to adoption of the current generation of vectors lies in their limited efficiency, resulting in limited transduction of CNS target cells. To address this limitation, researchers have bioengineered fit-for-purpose AAVs with improved CNS tropism and tissue penetration. While the preclinical assessment of these novel AAVs is primarily conducted in animal models, human induced pluripotent stem cell (hiPSC)-derived organoids offer a unique opportunity to functionally evaluate novel AAV variants in a human context. In this study, we performed a comprehensive and unbiased evaluation of a large number of wild-type and bioengineered AAV capsids for their transduction efficiency in hiPSC-derived brain organoids. We demonstrate that efficient AAV transduction observed in organoids was recapitulated *in vivo* in both mouse and non-human primate models after cerebrospinal fluid (CSF) delivery. In summary, our study showcases the use of brain organoid systems for the pre-screening of novel AAV vectors. Additionally, we report data for novel AAV variants that exhibit improved CNS transduction efficiency when delivered via the CSF in *in vivo* preclinical models.

INTRODUCTION

Adeno-associated virus (AAV) are small, non-pathogenic viruses that can be engineered into recombinant AAV (rAAV) vectors and used to deliver specific genetic cargo to target cells.[1–3] rAAVs have become a popular tool for genetic engineering and gene therapy due to their non-pathogenic nature and ability to efficiently transduce a wide variety of cell types, including both actively dividing and post-mitotic cells. The use of rAAVs for clinical gene delivery offers the promise of treatment, or even curing, thousands of genetic disorders, with the successes highlighted by recent market approval of multiple gene therapies using rAAVs, such as AAVrh74-based delandistrogene moxeparvovec (Elevidys), AAV5-based etranacogene dezaparvovec (Hemgenix), AAV2-based voretigene neparvovec (Luxturna), and AAV9-based onasemnogene abeparvovec (Zolgensma).[4–7] Furthermore, more than 130 clinical trials using AAV are currently ongoing, highlighting rAAV as a key translational gene delivery tool. It is important to note that many of these ongoing AAV-based clinical trials are directed at chronic neurological diseases, including neurodegenerative diseases (Parkinson's disease, Alzheimer's disease, and Huntington's disease),[8,9] lysosomal storage diseases,[10] and brain tumors.[11] The success of these and future trials relies on access to AAVs that enable highly efficient and specific delivery of clinical cargo to the specific target cells, highlighting a critical need for high efficiency central nervous system (CNS)-targeting AAV variants.

Importantly, the successes of AAV-based therapies targeting the CNS depend on the ability to overcome a number of significant challenges, including (1) the high vector doses required for systemic intravenous

Received 23 February 2024; accepted 25 June 2024;
https://doi.org/10.1016/j.omtn.2024.102264.

[8]Present address: INSERM U951, Généthon, Evry, 91000, France
[9]Present address: Galicia Sur Health Research Institute (IIS Galicia Sur), SERGAS-UVIGO, Vigo, Spain
[10]Present address: SpliceBio, Baldiri Reixac 10-12, 08028 Barcelona, Spain
Correspondence: Leszek Lisowski, Translational Vectorology Research Unit, Children's Medical Research Institute, Faculty of Medicine and Health, The University of Sydney, Westmead, Australia.
E-mail: llisowski@cmri.org.au

Published by Elsevier Inc. on behalf of The American Society of Gene and Cell Therapy.
This is an open access article under the CC BY license (http://creativecommons.org/licenses/by/4.0/).

(i.v.) administration to achieve therapeutic efficacy, which can lead to undesirable off-target tissue transduction and associated toxicity in peripheral organs[12–14]; (2) the presence of circulating pre-existing neutralizing antibodies in a large proportion of the patient population, which recognize and neutralize AAVs, lowering their clinical efficacy[15,16]; and (3) the presence of the protective blood-brain barrier (BBB), which limits CNS access after i.v. administration.[17] The BBB is a biological barrier that plays a key role in brain homeostasis by strictly controlling the exchanges between the blood and the brain compartments and protecting the CNS from viral and bacterial infections. As such, the BBB poses a significant obstacle to the entry of AAV into the CNS.

Therapeutic vector administration through local routes, such as intraparenchymal administration or injection into the cerebrospinal fluid (CSF) (e.g., intracerebroventricular [ICV], intracisterna magna [ICM], or lumbar intrathecal injections), allows to bypasses these challenges to CNS gene delivery, and thus offers benefits compared with i.v. administration.[18–20] Specifically, local administration has been shown to enhance the target tissue transduction, limit off-target transductions, and decrease the vector doses required to achieve clinical benefits, consequently reducing the risk of neutralization by antibodies, immunogenic reactions, or toxicity.[21]

A central feature of AAV vectors is their capsid-driven tissue- and cell-type tropisms.[22] The self-assembly of the capsid from the three structural proteins (VP1, VP2, and VP3) encoded by the viral *cap* gene can be leveraged in bioengineering strategies to modulate tissue tropisms, alter biorecognition, and enhance the performance of AAV vectors. Recent advances in rAAV bioengineering strategies, including rational design and directed evolution, have improved our ability to design and select novel AAV variants with desired properties.[23] Importantly, the choice of the biological model used for vector bioengineering and validation has a significant and direct influence on the fitness and thus therapeutic efficacy of the selected vector. Clinical translation of novel therapeutics depends on our ability to evaluate vector performance and safety in the most biologically and clinically predictive preclinical models. Lessons from previous AAV capsid selections in rodent models have shown that variants engineered using such models have thus far failed to translate in Old World Monkey non-human primates (NHPs)[24,25]; therefore, they do not contribute to progress toward clinically applicable vectors.

In recent years, induced pluripotent stem cell (iPSC)-derived brain organoids have emerged as a powerful tool for studying human brain development and model for human diseases. Brain organoids are three-dimensional (3D) assemblies of neurons that recapitulate many aspects of brain development and cyto-architecture.[26,27] As a result, human brain organoids may represent a valuable platform to investigate AAV capsid transduction efficiency, toxicity, and cell tropism within the human CNS.[28–30] While brain organoids lack the presence of a BBB, they are limited to predicting the performance of AAV capsid variants upon direct brain injection. Nonetheless, their value in understanding AAV-mediated gene delivery in a human-like neural setting is indispensable.

In this study, we used two types of human organoids—dorsal cortical organoid[31] previously described by our group and the commonly used cerebral brain organoid described by Lancaster et al.[26]—to perform parallel high-throughput functional assessment of a large set of natural and bioengineered AAV variants. We identified 10 AAV variants that demonstrated high transduction efficiencies in these preclinical models. After a detailed analysis of individual variants in the organoid models, we performed further *in vivo* validation in both mouse and NHP models following ICV and ICM administration, respectively. Two bioengineered variants, AAV-SYD12 and AAV2-M1, emerged as the top-performing variants with increased biodistrubution throughout both mouse and NHP brains. Interestingly, AAV-SYD12 was originally bioengineered for improved transduction of primary human hepatocytes *in vivo*, while AAV2-M1 was selected for its superior ability to transduce primary human retina.[32,33]

Our data support the hypothesis that AAV capsid screening in human brain organoids holds great promise for the identification of promising variants to enter clinical evaluation. Notably, this study represents the first evaluation of AAV capsids in animal models that were initially screened in human brain organoids. The consistently high transduction efficiency exhibited by our top AAV variants in both organoid and animal models underscores the enhanced clinical translatability of these AAV capsids.

RESULTS

Functional evaluation of AAV capsids in cortical and cerebral organoids

Several AAV variants have been reported as effective for gene delivery targeting the CNS across various pre-clinical animal models and human clinical trials. To directly compare the performance of these CNS-tropic AAVs in human brain organoids, we created a bespoke 13 AAV CNS Testing Kit, as previously described,[34] which contained an equimolar mix of 13 uniquely barcoded AAV variants (Table S1).

To prepare the 13 AAV CNS Testing Kit, each AAV variant was used to package two barcoded single stranded (ss)CMV-eGFP-BC-WPRE-BGHpA expression cassettes that allows for high-throughput next-generation sequencing (NGS)-based analysis of vector performance at the cell entry (DNA) and transgene expression (RNA/cDNA) levels.[34]

Both cortical and cerebral human organoids were transduced with the 13 AAV CNS Testing Kit at a dose of 2.5×10^{10} vg/organoid. Cortical organoids were transduced at day 103 ($n = 2$ organoids, $N = 2$ differentiation batches, 4 organoids analyzed in total), and whole brain organoids at day 120 ($n = 3$ organoids, $N = 1$ differentiation batch, 3 organoids analyzed in total) of culture. The organoids were harvested two weeks after transduction and DNA and RNA were extracted

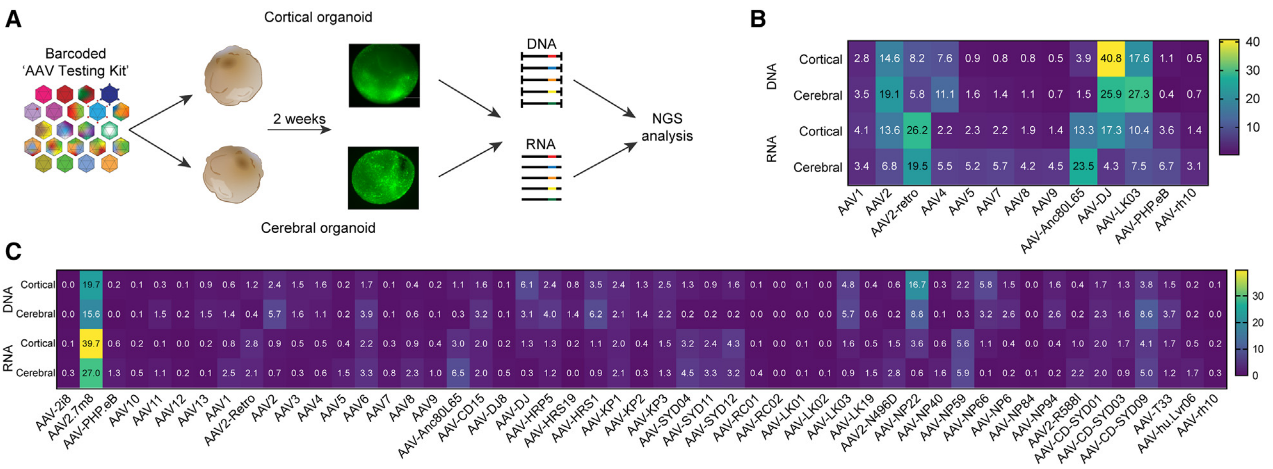

Figure 1. Assessment of AAV transduction in iPSC-derived cortical and cerebral organoids

(A) AAV Testing Kit workflow. The kit was used to transduce cortical and cerebral organoids before DNA and RNA was obtained and processed for NGS. (B and C) Percentage of NGS reads mapped to each barcoded AAV variants (n = 2 barcodes/capsid) in cortical and cerebral organoids at the DNA (cell entry, physical transduction) and cDNA (expression, functional transduction) levels, normalized to the pre-injection mix (B) for the 13 AAV CNS Testing Kit and (C) the 51 AAV Testing Kit.

for analysis. We used PCR to amplify the 150-bp region of the rAAV genome that contained the unique barcode (BC) for vector identification. All samples were submitted for NGS analysis to establish the relative performance of individual AAV variants at the level of cell entry (DNA) and transgene expression (RNA) (Figure 1A).

At cell entry, the top three performing capsids were AAV-DJ, AAV-LK03, and AAV2 (Figure 1B). Remarkably, these three vectors contributed 72% and 73% of all the DNA reads in cortical and cerebral organoids, respectively (Figure 1B). Interestingly, AAV9, AAV-rh10, and AAV-PHP.eB, renowned for their capability to traverse murine BBB and transduce brain cells,[35–37] were the least abundant variants in both organoids at the DNA level. At the RNA level, the top-performing vectors differed between the cortical and cerebral organoids. For cortical organoids, AAV2-retro was the top-performing variant (26% of RNA reads), followed by AAV-DJ, AAV-LK03, and AAV2 (17%, 13%, and 10% of RNA reads, respectively). AAV2-retro was also a top performer in the cerebral organoids, along with AAV-Anc80L65, contributing to 20% and 24% of RNA reads, respectively. A human liver-tropic vector, AAV-LK03,[40] unexpectedly demonstrated efficient performance at cell entry and transgene expression in both organoid models.

Encouraged by the outcomes of this initial small-scale AAV screen, and the noteworthy absence of documented large-scale AAV screenings using brain organoid models,[28–30] we next thought to broaden our investigation with an increased number of variants. A total of 51 AAV capsids were included in the next iteration of the Kit, with the 51 AAV Testing Kit, including wild-type (WT) AAV serotypes 1–12, and bioengineered capsids (Table S1). Using this kit, we administered a total dose of 5×10^{10} vg/organoid (equivalent to 1×10^{9} vg/variant) in both the cortical and cerebral organoids. Two weeks later, the organoids were collected for DNA and RNA extraction and NGS-based quantification of the BC signal to determine the top-performing variants.

Strikingly, AAV2.7m8, which was not part of the initial study, exhibited the greatest efficiency for both types of brain organoids at both cell entry (cortical organoid, 20% of reads; cerebral organoid, 16% of reads) and expression (cortical organoid, 40% of reads; cerebral organoid, 27% of reads) levels (Figure 1C). It is noteworthy that this variant has already been reported by our group, and others, to perform very well in immortalized cell lines, as well as in primary cells and organoids *in vitro*.[33,34,38] Similarly, several next-generation bioengineered capsids, such as AAV-SYD04, AAV-SYD11, AAV-SYD12, and AAV-NP59, which showed superior transduction efficiency in primary human hepatocytes *in vivo* in a xenograft model of human liver,[32,39] were found to transduce cerebral organoids with high efficiency (Figure 1C).

Novel retinal capsid variants identified as top performing in brain organoids

The high performance of AAV2.7m8 during our 51 AAV Testing Kit screening was of special interest, considering that brain organoids are derived from retinal confluent cultures of PSCs,[31] and AAV2.7m8 is a peptide display variant developed to target the murine outer retina.[40] Based on this observation, we were prompted to assess the efficiency of 20 novel capsid variants that were recently selected by directed evolution in primary human retinal explants, and then further validated in retinal organoids,[33] for their efficiency of brain organoid transduction. Termed the 25 AAV Retina Kit, this rAAV mix contained the 20 novel retinotropic variants, as well as 5 benchmark rAAVs (AAV2, AAV8, AAV13, AAV2.7m8, and AAV-Anc80L65). To assess these variants, the 25 AAV Retina Kit was used to transduce both cortical and cerebral organoids at a dose of 2.5×10^{10} vg/organoid (equivalent to 1×10^{9} vg/variant). As with the previous studies, cells were

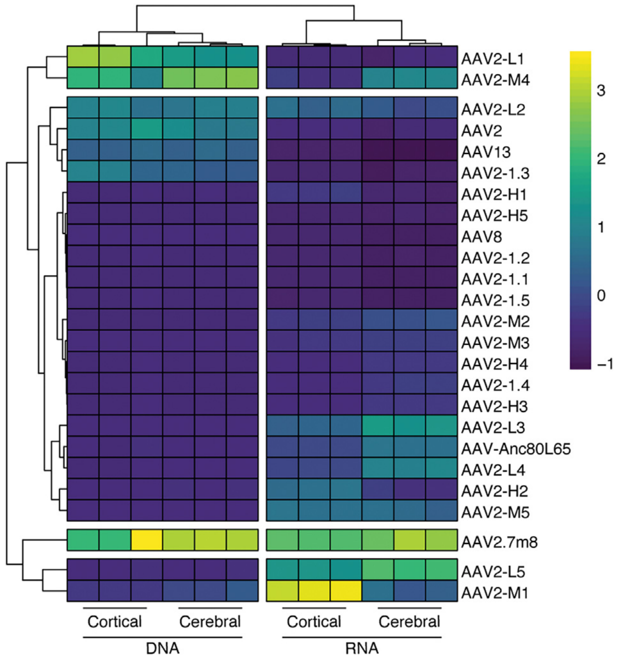

Figure 2. Performance of AAV retinal-targeted variants in cortical and cerebral organoids

Percentage of NGS reads mapped to each barcoded AAV capsid variant (n = 2 barcodes/capsid) from cortical and cerebral organoids harvested 14 days after transduction. Data represent percentage of total NGS reads from DNA (cell entry/physical transduction) and RNA (transgene expression/functional transduction). Percentages are normalized to the pre-injection mix.

collected 2 weeks after transduction for NGS analysis of vector efficiency at the DNA and RNA levels (Figure 2). Consistent with the results of the 51 AAV Testing Kit study, AAV2.7m8 outperformed all the other variants with AAV2-L1 and AAV2-M4 being the second and third most effective capsids at cell entry (DNA).

At the RNA level, AAV2.7m8 and two novel retinotropic variants, AAV2-L5 and AAV2-M1, showed the highest efficiency at functional transduction. In cerebral organoids, AAV2.7m8 showed the highest efficiency, followed by AAV2-L5, while in cortical organoids AAV2-M1 was the best performing capsid followed by AAV2.7m8 and AAV2-L5.

AAV2.7m8, AAV2-L5, AAV2-M1, and AAV-SYD12 show specificity for human neurons

We next aimed to validate the NGS data by performing a detailed evaluation of individual selected lead candidates identified in our three AAV Testing Kit screens. Based on data presented above (Figures 1 and 2), we chose 10 top-performing AAV variants: AAV2, AAV2-retro, AAV-DJ, AAV-Anc80L65, AAV-LK03, AAV-SYD12, AAV-NP59, AAV2-L5, AAV2-M1, and AAV2.7m8. These variants were used to package a ssAAV cassette encoding an eGFP fluorescent protein under the control of the ubiquitous CMV promoter (ssAAV-CMV-eGFP-WPRE-BGHpA).

Cortical organoids cultured for 120 days were transduced with individual variants at a dose of 1×10^{10} vg/organoid. The experiment was performed in three independently differentiated cortical brain organoids (n = 1 organoid; N = 3 differentiation batches, 3 organoids analyzed in total) per AAV, and expression of eGFP was assessed two weeks after transduction (Figure 3A). As was anticipated based on the NGS-based kit studies, vector-driven eGFP expression was evident for all variants assessed. eGFP expression was subsequently quantified using ImageJ software to measure average fluorescence for multiple images per organoid. While the data revealed no significant differences across the 10 AAV vectors tested (Figures 3A and 3B), AAV2.7m8, AAV2-L5, and AAV2-M1 were the best performing variants (Figure 3B).

We next aimed to determine the specific cell tropisms of the three top AAV variants (Figures 3C and S3A). Co-staining with the neuronal nuclear (NeuN) marker showed strong transduction of neurons for all three variants, with AAV2.7m8 transducing 34%, AAV2-L5 transducing 35%, and AAV2-M1 transducing 35% of NeuN-positive cells (Figure 3D). In contrast, eGFP expression was detected in few GFAP-positive cells for all three variants (Figure S3A).

The 10 lead AAV performers were also evaluated in cerebral organoids (Figure 4). Day 120 organoids were transduced with individual variants at a dose of 1×10^{10} vg/organoid. The experiment was performed in two independent batches of differentiation (n = 1 organoid; N = 2 differentiation batches, 2 organoids analyzed in total) and expression of eGFP was assessed 2 weeks later by immunofluorescence (Figure 4A). Although no significant differences in transduction efficiency were observed between the assessed variants, clear trends were observed based on fluorescence intensity. AAV2-L5 and the human liver tropic variant, AAV-SYD12, were found to lead the strongest eGFP signal (Figure 4B). In contrast, AAV-NP59 and AAV-LK03, which are known for efficient transduction of human primary hepatocytes *in vivo*, showed weaker expression. Similar to the previous results in cortical organoids, AAV2.7m8 also transduced cerebral organoids with high efficiency, similar to AAV-Anc80L65 (Figure 4B).

Based on these result, the top three variants, AAV2.7m8, AAV2-L5, and AAV-SYD12, were selected for further characterization of cell specificity using immunofluorescence analysis (Figures 4C and S3B). All three variants transduced neurons with differing efficiencies, with AAV2.7m8 and AAV-L5 showing the highest number of eGFP transduced NeuN-positive cells (Figure 4C). eGFP expression was also detected in GFAP-positive cells for all three variants (Figure S3B).

AAVs identified in human brain organoids enable widespread transduction in adult rodent brains

Our next objective was to validate the potential of the lead candidates for their ability to drive transgene expression *in vivo*. To this end, C57BL/6J mice were individually injected with the top four performing variants, AAV2.7m8, AAV2-L5, AAV2-M1,

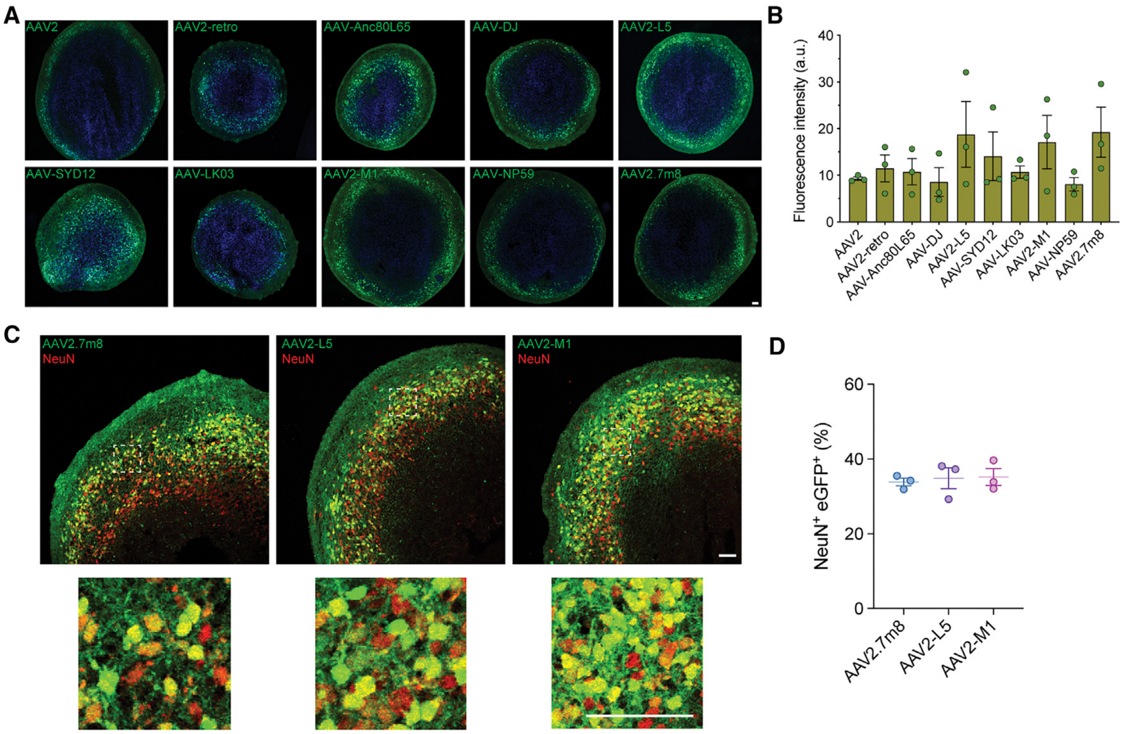

Figure 3. Comparison of transduction efficiency of cortical organoid models with selected top AAV variants

(A) Representative images of cortical organoids transduced with the indicated AAV variants encoding ssAAV-CMV-eGFP-WPRE-BGHpA cassettes (n = 1 organoid; N = 3 differentiation batches, 3 organoids analyzed in total, dose: 1 × 10^{10} vg/organoid, 2 weeks of expression). Green: vector-encoded eGFP; blue: DAPI (nuclei). Scale bars, 50 μm. (B) Quantification of the mean eGFP fluorescence intensity per cell transduced with indicated vectors. Data are mean ± SEM. Individual data points represent the average of two to six non-overlapping images per organoid batch. (C) eGFP reporter expression in cortical organoids after transduction with AAV2.7m8, AAV2-L5, and AAV2-M1 variants. Staining with NeuN was used to identify neurons (n = 1 organoid; N = 3 differentiation batches, 3 organoids analyzed in total, dose: 1 × 10^{10} vg/organoid, 2 weeks of expression). Dotted square indicates enlarged area. Red: neurons; green: vector-encoded eGFP. Scale bars, 50 μm. (D) Percentage of eGFP-positive neurons in cortical brain organoids transduced with AAV2.7m8, AAV2-L5, and AAV2-M1 (n = 1 organoid; N = 3 differentiation batches, 3 organoids analyzed in total, dose: 1 × 10^{10} vg/organoid, 2 weeks of expression). Data are mean ± SEM. Individual data points represent the average of five non-overlapping images per organoid batches.

and AAV-SYD12, encoding ssAAV transgene cassettes (ssAAV-CMV-eGFP-WPRE-BGHpA). Mice received bilateral ICV injections at a dose of 1.1 × 10^{10} vg/AAV/animal and were harvested 3 weeks after vector administration for transgene expression analysis using immunohistochemistry (Figure 5A).

ICV injections of AAV2.7m8 (n = 2 mice), AAV-L5 (n = 4), AAV-SYD12 (n = 4), and AAV2-M1 (n = 3) vectors led to eGFP expression for all vectors assessed (Figure 5). eGFP fluorescence was particularly abundant in the dentate gyrus of the hippocampus, but was also consistently evident in brain regions immediately adjacent to the ventricular system, such as the ventral cingulate cortex, lateral septal nucleus, striatum, and cerebellum. By applying a threshold to microscope images and measuring the number of pixels that exceeded that threshold (Figure S2), we found that eGFP expression was more widespread after AAV2-M1 administration compared with AAV2.7m8, AAV2-L5, and AAV-SYD12 (Figure 5B), covering an area of 4.6 ± 0.9 × 10^6 μm^2 per section compared with 0.7 ± 0.2 × 10^6 μm,^{2} 1.3 ± 0.3 × 10^6 μm^2, and 1.4 ± 0.4 × 10^6 μm^2, respectively for the other vectors (one-way ANOVA, p = 0.0028; Tukey's multiple comparisons test: AAV2.7m8 vs. AAV2-M1, p = 0.0064; AAV2-L5 vs. AAV2-M1, p = 0.0053; AAV2-M1 vs. AAV-SYD12, p = 0.0068). Surprisingly, given the bright appearance of the most densely labeled regions, no significant differences in eGFP fluorescence were observed (Figure 5C).

eGFP expression mediated by AAV2.7m8, AAV-L5, and AAV-SYD12 was essentially restricted to the hippocampus, with a small number of neurons observed in the deep layers of the somatosensory cortex immediately adjacent to the lateral ventricle and in some cases scattered eGFP expressing neurons that extended through the lateral septal nucleus and striatum (Figure 5D). In contrast, eGFP expression driven by AAV2-M1 was consistently more extensive, labeling all of the above structures and, in addition, significant numbers of Purkinje neurons in the outermost layer of the cerebellum as well as conspicuous fiber bundles within the hypothalamus and medulla (Figure 5E). In addition, ICV administration of AAV2-M1 led to significant labeling of fibers, but not cell bodies, within the dorsal spinal cord and cuneate nucleus, with a lesser projection extending to the deeper layers of the spinal cord (Figure 5E). This distribution is consistent with central terminals of

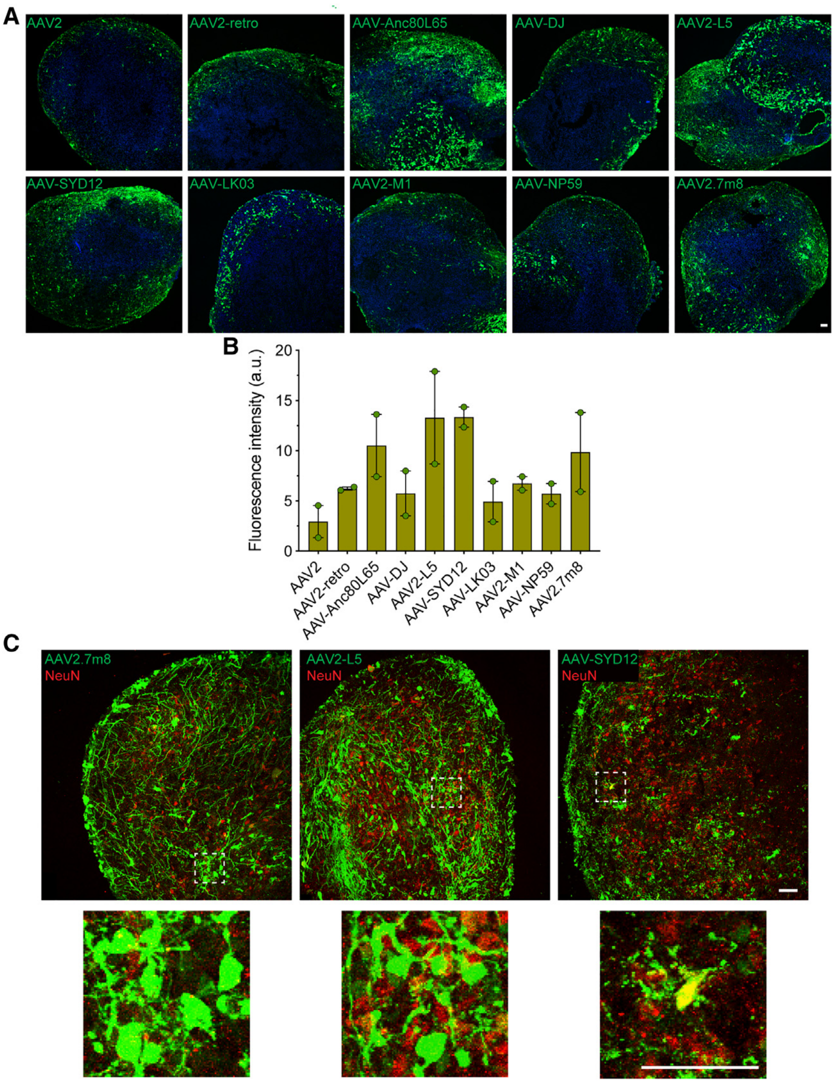

Figure 4. Comparison of transduction efficiency of cerebral organoid models with selected top AAV variants

(A) Representative images of cerebral organoids transduced with the indicated AAV variants encoding ssAAV-CMV-eGFP-WPRE-BGHpA expression cassettes (n = 1 organoid; N = 2 differentiation batches, 2 organoids analyzed in total, dose: 1×10^{10} vg/organoid, 2 weeks of expression). Green: vector-expressed eGFP; blue: DAPI (nuclei). Scale bars, 50 μm. (B) Quantification of the mean eGFP fluorescence intensity per cell transduced with indicated vectors. Data are mean ± SEM. Individual data points represent the average of three non-overlapping images per organoid batches. (C) eGFP reporter expression following transduction with AAV2.7m8, AAV2-L5, and AAV-SYD12 variants. Staining with NeuN was used to identify neurons (dose: 1×10^{10} vg/organoid, 2 weeks of expression). Dotted square indicates enlarged area. Red: indicates neurons; green: vector-expressed eGFP. Scale bars, 50 μm.

somatic primary afferent neurons. Unfortunately, no dorsal root ganglia (DRG) were retained; therefore, definitive identification of AAV2-M1-transduced DRG neurons was not possible.

AAVs identified in human brain organoids transduce NHP brain with high efficiency

One major drawback of bioengineered AAV variants has been their limited capacity to retain their CNS-tropism across multiple animal models.[24,25,41] Beyond ethical concerns surrounding the use of NHPs in biomedical research, the limited availability and high costs associated with their use can present substantial barriers, particularly for initial vector development and functional proof-of-concept studies. For this reason, the use of AAV testing kits provides a scientifically powerful and more ethical alternative strategy allowing for the assessment of a higher number of AAV variants in parallel within a single animal and avoiding animal variability. With this in mind, we produced an NHP CNS 20 AAV Kit containing 20 AAV capsids, including 3 WT variants and 17 capsid-engineered variants (including 9 heparan sulfate proteoglycan [HSPG] de-targeted variants) (Table S1). We included the HSPG-detargeted variants, as previous research has demonstrated that reduced binding to HSPG facilitated widespread transduction throughout the mouse brain.[42,43]

We performed an *in vivo* evaluation of the NHP CNS 20 AAV Kit in a young adult cynomolgus monkey following ICM infusion. The animal received a total AAV dose of 1.4×10^{13} vg, equivalent to 7×10^{11} vg/variant. Four weeks after injection, brain, spinal cord, and liver tissues were harvested and processed for downstream analysis (Figure 6A). Samples were first analyzed for vector copy number (VCN) using droplet digital PCR (ddPCR) (Figure 6B). Among all the samples assessed, the highest VCN was detected in both the cervical and thoracic sections of the spinal cord, quantified as 12.96 vg/diploid genome (vg/dg) and 11.32 vg/dg, respectively. In the various brain structures assessed, the highest VCN was detected in the pons (0.68 vg/dg), occipital lobe (0.53 vg/dg), temporal lobe (0.29 vg/dg), putamen (0.24 vg/dg), and caudate (0.15 vg/dg). The VCN was notably lower in the thalamus (0.07 vg/dg), hypothalamus (0.10 vg/dg), hippocampus (0.05 vg/dg), cerebellum (0.03 vg/dg), medulla (0.09 vg/dg), parietal lobe (0.06 vg/dg), and frontal lobe (0.09 vg/dg). Of note, vector genomes were detected in the liver (2.63 vg/dg), a reading that is 10-fold higher than what was detected in the brain (Figure 6B).

In addition, we analyzed the relative vector performance in 12 brain regions and 3 sections of the spinal cord, both at the vector uptake (DNA) and transgene expression (RNA/cDNA) levels using NGS (Figures 6C, 6D, and S4). While the data were obtained from a single NHP, NGS analysis indicated that AAV-CD-SYD09, an

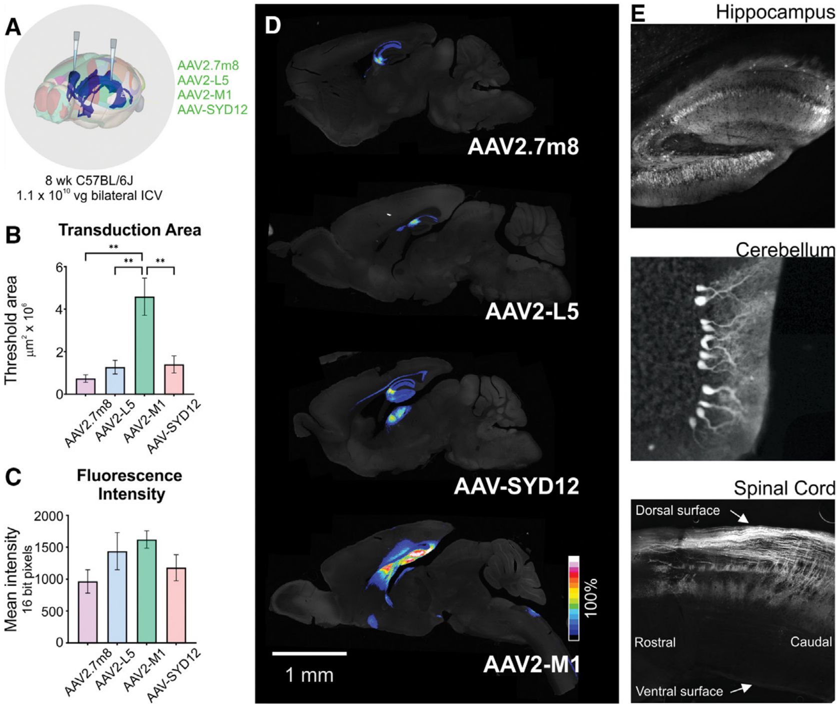

Figure 5. ICV injections in adult mice show superior transduction ability and vector spread of AAV vectors identified in brain organoids

(A) Graphical illustration depicting bilateral ICV injections of eGFP-encoding AAV2.7m8, AAV2-L5, AAV2-M1 or AAV-SYD12 into adult mice (1.1×10^{10} vg dose/mouse). Three weeks after injection, animals were sacrificed and brains were analyzed for eGFP expression. (B) Mean transduction area and (C) eGFP fluorescence intensity averaged across one hemisphere/mouse ($n = 2$ mice AAV2.7m8, $n = 4$ AAV2-L5, $n = 3$ AAV2-M1, $n = 4$ AAV-SYD12). Data are mean ± SEM. $^{**}p \leq 0.01$. Statistical significance was calculated using a one-way ANOVA with Tukey 's multiple comparisons test. (D) Representative sagittal brain sections showing eGFP transduction, normalized to AAV2-M1 expression. Scale bar, 1 mm. (E) Representative images illustrating AAV2-M1-driven eGFP expression in region of interest. Note: contrast and intensity of (E) have been optimized to discriminate features of interest.

AAV6-related serotype, transduced both the brain and the spinal cord at the highest efficiency based on DNA reads, presenting as the top-performing variant in the brainstem and cerebellum regions. These results indicate the ability of AAV-CD-SYD09 to enter cells at a high efficiency. In the other brain regions assessed, the variants that achieved the greatest cell entry efficiency included the HSPG de-targeted variant, AAV2-N582S, in the cerebral cortex, and AAV2.7m8 along the spinal cord sections (Figures 6C and S4). In terms of transgene expression, AAV2-M1, AAV-SYD12, and AAV-CD-SYD09 were the highest performing variants throughout all the brain and spinal cord regions (Figures 6C and S4). AAV2-M1 and AAV-SYD12 were the most efficient in the cerebral cortex, cerebellum, and spinal cord, while AAV-CD-SYD09 was the top-performing variant in the diencephalon region (Figures 6D and S4).

While the vectors were not delivered via systemic i.v. infusion, the ability of the vectors to leak into the peripheral circulation and transduce the liver was of concern,[44,45] as liver toxicity is frequently reported in preclinical and clinical studies.[46] Analysis of the NHP liver samples revealed that AAV-CD-SYD09, AAV2-N582S, AAV2.7m8 and AAV9 were the most represented variants are the DNA level (cell entry) (Figure S4), but no transgene expression could be detected at the RNA/cDNA level.

While the NGS analysis provides a prediction of the relative efficiency of the different serotypes, further investigations are needed to characterize the top-performing variants in NHPs. Overall, our results illustrate that AAV2-M1 and AAV-SYD12 are cross-species compatible vectors capable of efficient transduction of the CNS in the murine, human brain organoids, and NHP models.

DISCUSSION

Developing safe and efficient AAV-based therapeutics for disorders affecting the CNS faces several challenges, including constrained BBB penetration and limited biodistribution within the CNS after i.v. administration. In addition, the lack of adequate preclinical models that facilitate the translation of AAV development from bench to bedside is a critical challenge that needs to be overcome if novel AAV-based therapeutics are to reach their full clinical potential. Therefore, in this study, we used stem cell-derived cortical and cerebral organoids to evaluate the performance of natural and bioengineered AAV vectors to identify AAV candidates for potential human applications. Through this approach, we successfully identified 10 AAV capsids that exhibited a high level of transduction efficiency within the human organoid models. After Following further *in vivo* evaluation in mouse and NHP models, two variants, AAV2-M1 and AAV-SYD12, were shown to exhibit substantially increased spread and efficiency in the CNS after CSF delivery.

Our AAV testing kit approach[34] was first used to perform a large screen of previously described and bioengineered AAV variants in human brain organoid models. Brain organoids offer a robust approach for functional evaluation of AAV-based therapeutics targeting CNS indications.[47] This is particularly pertinent due to the difficulties of obtaining relevant, viable human brain tissue samples (e.g., from brain biopsies), and the obstacles related to their maintenance in culture and lack of scalability. Moreover, few studies have documented the use of AAV vectors in human organoids with the aim of identifying efficient capsids for translational outcomes and high transgene expression within the human brain.[28–30] In our study, AAV2.7m8 was identified as a top performing variant across both cortical and cerebral organoids. This is in line with a previous report

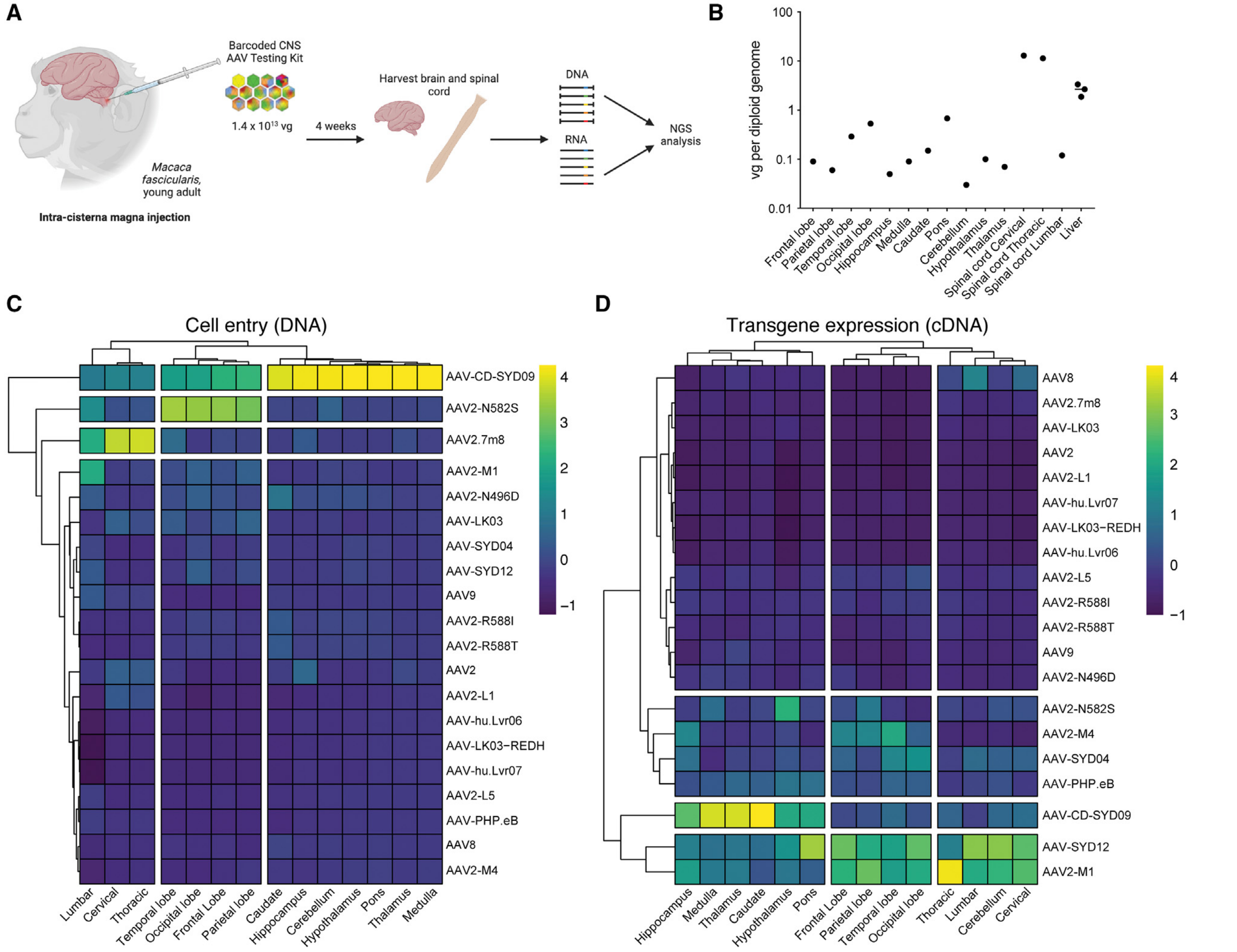

Figure 6. Functional evaluation of AAV vectors in NHPs following intra-cisterna magna injection

(A) Graphical illustration depicting barcoded AAV kit delivery in NHPs to study transduction across the CNS at 4 weeks after delivery. $N = 20$ AAV variants were mixed at an equimolar ratio (known as the NHP CNS 20 AAV Kit) and injected ICM at a total dose of 1.4×10^{13} vg in one cynomolgus NHP (*M. fascicularis*, young male adult). At 4 weeks after injection, the brain, spinal cord, and liver of the animal were harvested for downstream analysis. (B) VCN analysis in tissue samples from the indicated brain and spinal cord regions and the liver. (C and D) Heatmap plots representing the transduction efficiency of each AAV variant within individual regions of the brain and spinal cord, expressed as the percentage of NGS reads. The analysis was performed at (C) the DNA (cell entry) and (D) the RNA (transgene expression) levels.

from Duong et al., where AAV2.7m8 was found to drive the highest eGFP transgene expression across cortical neurons.[38] Additionally, research using cerebral organoids identified AAV-DJ and AAV-Anc80L65 as top-performing vectors.[48] Our data obtained by using the 13 CNS AAV Testing Kit affirm the remarkable transduction efficiency of these two variants, validating their ability to effectively target and transduce brain organoids. The experiments with the 51 AAV Testing Kit revealed that bioengineered AAV-SYD12 and AAV-NP59 capsids, developed for targeting human hepatocytes, were among the best performing variants in both cortical and cerebral organoids. Finally, the NGS data obtained with the 25 AAV Retina Kit identified AAV2-M1 and AAV2-L5 as top-performing variants in cortical and cerebral organoids, respectively.

Further, in-depth characterization of these top AAV variants revealed their capacity to efficiently transduce neuronal cells while transducing GFAP-expressing astrocytes with very low efficiency, highlighting their possible potential for targeting neurons in the human brain.

While brain organoids offer a more complex model compared with traditional 2D *in vitro* systems, they still do not fully recapitulate the complexity of *in vivo* models due to their limited size and heterogeneity. Therefore, we next assessed the top-performing variants *in vivo* using a murine model. In all cases, ICV injection of vectors led to eGFP expression in neurons immediately adjacent to the ventricular system, with the hippocampus and ventricle-adjacent parts of the ventral cortex containing the majority of transduced neurons.

The bioengineered retinotropic variant, AAV2-M1, showed improved transduction, labeling neurons in regions of the brain remote from the injection site (e.g., cerebellar Purkinje neurons) to an extent not seen with the other vectors tested. However, no penetration of any vector into the deep parenchyma of the brain was ever seen in the current study.

The approach used to quantify the distribution of eGFP fluorescence is subject to a number of caveats. First, the vectors tested drove expression of eGFP throughout the cytoplasm, including the cell body but also its dendrites, axons, and synaptic terminals. This means that fiber tracts originating in regions that contained a high number of transduced neuronal cell bodies would have contributed to calculations of area, perhaps leading to overestimation of transduction spread; for example, the major output bundle of the hippocampus, the fornix, is clearly visible in Figure 5D. Conversely, the detection of relatively faintly labeled fibers of passage following AAV2-M1 administration may also have led to underrepresentation of fluorescence intensity with the AAV2-M1 vector, as labeling in these regions fell under the threshold for detection for the other vectors tested. A better approach for quantification of the distribution of transduced neuronal cell bodies would have been to use a nuclear localization sequence to restrict GFP expression to the nucleus.

One interesting result of the current study is the observation that AAV2-M1 yielded extensive labeling of fibers and terminals innervating the dorsal spinal cord and medullary sensory relays. Although a number of brain regions project to the dorsal horn of the spinal cord to modulate ascending somatosensory inputs, we did not observe any labeled cell bodies in the brainstem nuclei thought to represent the major sources of such input, the rostral ventromedial medulla and locus coeruleus.[49,50] Neurons of the somatosensory[51] and anterior cingulate[52] regions of the cortex also project to the spinal dorsal horn, and all vectors effectively transduced cortical neurons adjacent to the ventricles, particularly those in the deeper layers of the somatosensory cortex. However, we observed no labeling of the medullary pyramids or pyramidal decussation, easily identifiable landmarks of the corticospinal tract in any experiment, and therefore tentatively speculate that the extensive labeling observed in the dorsal horn may reflect a tropism of AAV2-M1 for sensory neurons. The central projections of DRG neurons are bathed in CSF, and therefore could plausibly have been accessed by ICV injections. Definitive proof, in the form of eGFP-labeled peripheral nerves or DRG, was not collected.

To compare the results observed in organoids and mice to vector performance in large primates, we next performed an evaluation of 20 capsids after ICM delivery in NHPs. By assessing the top-performing variants across human organoids, mice, and an Old World Monkey, this multispecies approach was intended to address the long-standing challenge of translatability in the field of CNS AAV development. Indeed, ICM injection allows for the selective and localized targeting of neural tissues, including the CNS and associated structures.[19] AAV2-M1 was one of the top performing variants at the level of transgene expression throughout the NHP brain. These findings suggest that AAV2-M1's ability to transduce the CNS is maintained across species, positioning this novel capsid as a potential candidate for translational applications. Future research will be required to fully evaluate the performance of AAV2-M1 and characterize the cell type tropism in NHPs.

Interestingly, the AAV-SYD12 variant was found to perform with high efficiency within all the NHP brain structures. Of note, AAV-SYD12 is an HSPG de-targeted variant composed of capsid fragments from AAV2, AAV7, and AAV10. This observation is aligned with the fact that AAV2 variants harboring amino acid changes that decrease their HSPG binding been shown to have improved transduction and increased tissue penetration in the mouse brain.[42,43] Interestingly, however, other AAV2 mutants that do not bind HSPGs (e.g., AAV2-N496D, AAV2-R588T, AAV2-R588I, AAV-LK03-REDH, and AAV-hu.Lvr06)[53–55] have not exhibited the same functional improvements in the CNS. This could indicate that the enhanced biodistribution achieved by AAV-SYD12 in the NHP brain may be a result of an as-yet unidentified amino acid motif or region of the capsid. Alternatively, it is possible that a very specific range of HSPG affinity is required to achieve the optimal effect, with excessive or insufficient HSPG binding negatively impacting on vector's function. Further studies are needed to elucidate the mechanism of action of the AAV-SYD12 variant and to evaluate whether other AAV serotypes can be engineered in a similar manner to enhance transduction in the NHP brain.

In the NHP brain, we also observed the high performance of AAV-CD-SYD09 at both the cell entry and transgene expression levels. This variant was previously selected for improved transduction of human hematopoietic stem/progenitor cells and shares high homology with natural AAV6 serotype (98.6% identity, 10 amino acids differences).[56] This variant was also shown to have high efficiency at both cell entry and transgene expression in our human organoid screen (Figure 1C). Interestingly, previous data have demonstrated the high performance of AAV6 in brain organoids,[28,48] iPSC-derived cortical neurons, and *ex vivo*-isolated rat cortical neurons,[38] suggesting that AAV6 and AAV6-based variants may be efficient for therapeutic gene delivery applications when injected directly into the human brain. Moreover, following ICV injection in adult mice, AAV-CD-SYD09 displayed efficient targeting of the cerebellum (data not shown). Given the strong performance of AAV-CD-SYD09 in the NHP model, future studies, outside the scope of this report, will be conducted to characterize this variant in greater detail.

While a broad targeting CNS gene delivery approach may be beneficial for many neurological disorders, certain conditions require more precise targeting of specific brain regions affected by the disease. Of particular interest is specific targeting of the cerebellum, the primary site of pathology in disorders such as cerebellar ataxias, Friedreich's ataxia, and cerebellar hypoplasia.[57] Importantly, our data suggest that AAV2-M1 and AAV-CD-SYD09 effectively target the cerebellum. If the efficiency of these variants can be individually validated

in large mammal models, both variants could be considered ideal candidates for gene delivery in therapies aimed at treating cerebellar conditions. Use of these specialized variants would allow for targeted gene delivery to the cerebellum while maintaining high efficiency and specificity.

Additionally, the general approach of this cross-screening study are expected to offer guidance to researchers in the field of gene therapy. We have proposed a general workflow that will facilitate the execution of similar studies and aid in the selection of the most suitable AAV capsid candidate(s) for specific neurological conditions. Following selection of an indication, patient-specific organoids can be generated to assess AAV transduction efficiency. While WT brain organoids were used in our study, previous work has demonstrated the utility of patient-derived organoids in identifying optimal AAV serotypes.[29] This approach allows for testing within a human-relevant system and provides valuable insights into potential *in vivo* responses. As brain organoids still lack many important features of a human brain, *in vivo* animal models can be used to assess the AAV biodistribution and the choice of the optimal delivery route to ensure adequate vector exposure to the relevant, affected areas of the brain.

The study described herein identifies key bioengineered AAV capsids with an improved ability to transduce cells of the CNS, as assessed in human brain organoids, mice, and NHPs upon CSF delivery. The observed high transduction efficiency in both human organoids and animal models underscores their translational potential and prospective utility in gene therapy applications to treat diseases and acquired conditions of the CNS. Their efficacy across animal models also could negate the need for a surrogate AAV in support of preclinical studies, substantially enhancing their translatability and thus clinical value.

MATERIALS AND METHODS

Differentiation of PSCs into cortical organoids

Cortical brain organoids were generated using our previously described protocol.[31] Specifically, human iPSCs were maintained until 90%–95% confluent. The cells were cultured in Essential 6 Media (E6, Life Technologies) for 2 days (replaced fresh between days 1 and 2 of differentiation). On day 3 of differentiation, the media was replaced with a pro-neural induction media (PIM, composed of Advanced DMEM/F12, N2 supplement, L-Glut, non-essential amino acid and antibiotic-antimycotic). At around week 3–4, 3D rosettes containing organoids were observed throughout the plate and in close proximity to neuroretinal vesicles. The 3D cortical organoids were manually excised with 19G needles and moved onto a 60-mm well plates in retinal differentiation media (composed of DMEM, F12 Nutrient mix, B27-vitamin A, and antibiotic-antimycotic) and put on an orbital shaker at 85 RPM. At 6 weeks of differentiation, retinal differentiation medium was supplemented with 10% FBS, 100 pM Taurine (Sigma, Cat# T4871) and 2 mM Glutamax. Starting week 10, the cortical organoids were cultured in a cerebral differentiation medium as described in Lancaster and Knoblich[58] (composed of neurobasal medium, DMEM/F12, N2 supplement, insulin, Glutamax, MEM-NEAA, B-mercaptoethanol, B27 supplement) on an orbital shaker with speed set at 85 RPM. Cortical organoids were fed every 3–4 days.

Cerebral brain organoid differentiation culture

Cerebral brain organoids (Figure S1) were generated using a protocol adapted from Lancaster and Knoblich.[58] Specifically, a human iPSCs line (HPSI0214i-kucg_2) was dissociated into single-cell suspension using Accutase (Life Technologies, Cat# 00-4555-56) and resuspended at 9,000 cell/150 μL in E6 (Life Technologies) with 4 ng/mL basic fibroblast growth factor (bFGF) and 50 μM Rock inhibitor (RI; Y-27632 dihydrochloride, Tocris). On day 0 of differentiation, 9,000 cells were plated per well in low binding 96-well U-bottom plates (NunclonTM 44ector, Thermo Fisher SCientific). On day 2, one-half of the old media was replaced with 150 μL of E6 media supplemented with 4 ng/mL bFGF and 50 μM RI. On day 4, all media was replaced with E6 media without bFGF and RI. On day 6, healthy embryoid bodies (EBs) with a diameter of more than 500 μM and translucent edges were transferred to low binding 24-well ultralow attachment plates (Costar, Corning) in 500 μL PIM (composed of Advanced DMEM/F12, N2 supplement, L-glutamine, non-essential amino acid, and antibiotic-antimycotic). Two EBs were transferred per well of low binding 24-well plates. On day 8, an additional 500 μL PIM was added to each well. On day 10, EBs with neuroepithelia were selected and a maximum of 16 EBs were transferred per 60-mm dish. Five milliliters of cerebral differentiation medium (IDM-A; composed of Neurobasal medium, DMEM/F12, N2 supplement, insulin, Glutamax, MEM-NEAA, B-mercaptoethanol, B27 supplement without retinoic acid) as described in Lancaster and Knoblich[58] was added and incubated at 37°C. On day 12, old media was replaced with fresh IDM-A. On day 14, media was replaced with IDM+A media (composed of neurobasal medium, DMEM/F12, N2 supplement, insulin, Glutamax, MEM-NEAA, B-mercaptoethanol, and B27 supplement) and the dish was put on an orbital shaker with speed set at 85 RPM. Cerebral brain organoids were fed every 3–4 days.

AAV transduction of human brain organoids *in vitro*

AAV vectors ($1–5 \times 10^{10}$ vg/organoid) were added to a total volume of 375 μL using fresh cortical organoid differentiation medium (CODM) used to culture the cortical organoids. The organoids were then transferred to low binding 24-well plates (Costar, Corning, Cat# 3524), and the media was completely replaced with CODM containing the AAV vectors. Cortical and cerebral organoids were incubated in a standard incubator at 37°C for one-half of a day before adding another 625 μL of fresh media. After overnight culture at 37°C, the organoids and CODM/vector mixture were transferred to a 60-mm dish. The dish was topped up with 4 mL fresh CODM and put on an orbital shaker inside the incubator at 85 rpm at 37°C. After the initial 48 h, organoids were fed every other day. Organoids were harvested for NGS preparation or immunofluorescence 14 days after AAV exposure.

DNA and RNA extraction from organoids

DNA and RNA were isolated from the cell using the AllPrep DNA/RNA Mini Kit (Qiagen, Cat# 80204) following the manufacturer's instructions.

AAV vector production

All AAV vectors used in this study were produced in adherent HEK293T cells using polyethylenimine hydrochloride (PEI Max, 40,000, Polysciences, Cat# 49553-93-7) transfection of the single-stranded CMV-eGFP-barcode transgene cassette, as well as pRep2/CapX (X stands for the various capsid genes used in this study) and pAd5 Helper plasmids using previously described methods.[34] The resulting cell lysates and vector-containing media were processed to purify vector particles using iodixanol gradients, as previously reported.[59] Vector preparations were tittered (see AAV vector titration) individually and subsequently mixed at equimolar ratio to generate individual kits.

For the NHP CNS 20 AAV Kit, the cell lysates only (no media) were individually titered and subsequently mixed at equimolar vector ratio. The vector mix was then purified using a double cesium chloride gradient ultracentrifugation following the previously published protocol.[60]

AAV vector titration

AAV vector preparations were quantified by ddPCR (Bio-Rad) using EvaGreen supermix (Bio-Rad, Cat# 1864035) following the manufacturer's instructions and eGFP-specific primers (GFP-F: 5′-TCAAGATCCGCCACAACATC; GFP-R: 5′-TTCTCGTTGGGGTCTTTGCT) at a final concentration of 1 μM.

Mouse housing and ethics

Mouse experiments were approved by the Macquarie University Animal Ethics Committee and were performed in accordance with the Australian Code for the Care and Use of Animals for Scientific Purposes (8th edition, National Health and Medical Research Council, 2013). C57/BL6 mice were purchased from the Animal Resources Center in Perth, Western Australia, and group housed in the Central Animal Facility at Macquarie University in Techniplast cages under a 12-h light/dark cycle. Food and water were available *ad libitum*. Mice underwent a 1-week acclimatization period.

Stereotaxic injections

Mice were anesthetized with isoflurane (5% in pure medical O_2 for induction, 2%–3% maintenance). The dorsal surface of the head was shaved, and animals were provided with prophylactic analgesia (5 mg/kg carprofen subcutaneously, Norbrook Pharmaceuticals) and an antibiotic (10 mg/kg cephazolin intramuscularly [i.m.], AFT Pharmaceuticals). Mice were placed in a stereotaxic frame, on a heating mat, with internal temperature being monitored by a digital rectal thermometer (Harvard Apparatus) and maintained at ~37°C. A dorsal midline incision was made to expose the skull and hydrogen peroxide was used to clear away the connective tissue for better suture visualization. Bregma and Lambda were used to determine if the skull was in a flat position in the horizontal plane. Bilateral burr holes were made to expose the brain surface. A volume of 2.5 μL of vector was manually injected into each lateral ventricle (5 μL total volume; 1.1×10^{10} total vg/mouse/vector) using a Hamilton syringe (#701 series, 10 μL, 30G needle) mounted on a stereotaxic manipulator. The coordinates for the injection were 0.5 mm caudal to bregma, ±1 mm mediolateral, and 2.3 mm deep. Each injection was performed over the course of 10 min, positioned using a NeuroStar stereotaxic robot and StereoDrive software (NeuroStar). The syringe was left in place for 5 min before retraction. The skin was closed with silk sutures.

Mouse tissue collection and preparation

Animals were sacrificed 3 weeks after injection with an intraperitoneal injection of sodium pentobarbital (100 mg/kg, Virbac) and transcardially perfused with 0.9% heparinized saline followed by fixation with 4% (w/v in PBS) paraformaldehyde (PFA). Brains were extracted and stored in PFA overnight before being transferred to tris-phosphate buffered saline with 0.01% merthiolate.

Brains were sliced into 60-μm sagittal sections using a vibrating microtome (Leica VTI200ss, Leica Microsystems Pty Ltd.). For determining the spread of the vector, sections were mounted onto glass slides with DAKO mounting medium and cover slipped.

Immunofluorescence staining, microscopy, and image analysis

Sagittal sections from one hemisphere per brain were analyzed to survey the spread and transduction efficacy of ICV vector injections, with equal numbers of sections per animal. Slides were imaged using a 16-bit greyscale camera at 10× magnification with an Olympus VS200 Slidescanner, using the same exposure settings for all experiments. For quantification of eGFP expression, histograms were balanced using the highest pixel intensity observed on any image to determine the range of all other images, and images were downsampled to 25% using Zen software and exported into Fiji ImageJ. For each section, the distribution and intensity of eGFP fluorescence were measured using a custom ImageJ macro that created a mask over the areas of each section that exceeded an arbitrary fluorescence threshold that was determined empirically (Figure S2). The area of the mask and average intensity of pixels contained within it were quantified for each section.

Brain organoids were washed with PBS and fixed for 40–60 min in 4% PFA before incubation in 20% sucrose. Organoids were embedded in optimal cutting temperature compound and frozen in liquid nitrogen. Brain organoids were cryo-sectioned at 14 mm thickness. Cryosections were blocked in 5% serum in blocking solution (1% BSA in PBS with 0.1% Triton X-) for 2 h. Primary mouse anti-NeuN antibody (1:300, Merk Millipore, MAB377), and rat anti-GFAP (1:200, Sigma, Cat# 345860) diluted in blocking solution were incubated overnight at 4°C. Sections were washed with PBS and incubated with secondary antibody (Alexa Fluor 546, and 633 secondary antibodies) at room temperature for 2 h. Sections were counter-stained with DAPI. Images were captured on a Leica Stellaris 8 confocal microscope. eGFP-positive NeuN cells were manually counted using the FJII plugin Cell Counter.[61]

NHP housing and ethics

NHPs experiments were approved by the University of Navarra Animal Ethics Committee (CEEA 068/21) and by the Animal Welfare

Section of the Livestock Service of Government of Navarra. *Macaca fascicularis* originally from Vietnam were purchased from Bioprim and group housed in the Animal Facility at the University of Navarra in cages under a 12-h light/dark cycle. Food was given twice per day and water was available *ad libitum*. NHPs underwent a 40-day acclimatization period. Once the acclimatization period ended, animals were grouped by sex in separated rooms where they followed the same feeding regime. General health and welfare were assessed at least twice a day by the facility caretakers and veterinarians.

NHP work

Initial neutralizing antibody titer against AAV9 was 1:5, but to prevent the inhibitory effect of the potential presence of neutralizing antibodies against any of the other AAV variants present in the AAV kit before the injection, one young adult male (3.8 kg) *M. fascicularis* NHP was subjected to immunoadsorption (IA) to remove circulating IgGs as previously described.[62] Within the following 30 min after IA, the vector was infused into the cisterna magna. The animal was sedated with ketamine (7 mg/kg i.m., Bayern) and midazolam (0.6 mg/kg i.m., B. Braun). The surface above the cisterna magna—an area corresponding with the union of the spine and the base of the skull—was shaved, the NHP was placed in a stereotaxic frame, on a heating mat, with internal temperature being monitored by a digital rectal thermometer (Roche) and maintained at ~37°C. Due to the use of propofol intubation, oxygen flow was required (1–1.5 L/min). Povidone-iodine was used topically to disinfect the area of the puncture. A 1-mL syringe (BD, with a 25G needle) containing the AAV vector was loaded in a mechanical arm attached to the stereotaxic frame, the syringe was guided to the cisterna magna by the surgeon. To ensure that the needle was correctly located, a sample of CSF was removed. Subsequently, the AAV CNS Testing Kit solution (900 μL in PBS supplemented with 50 mM NaCl and 0.001% Pluronic F68 (Gibco, Cat# 24040-032) at a dose of 1.4×10^{13} vg was infused at a rate of 0.5 mL/min. Once the complete volume was injected the syringe remained 10 additional minutes to avoid reflux. Once the needle was removed the area was disinfected and the animal was allowed to recover.

Four weeks after vector injection, the animal was sedated with ketamine (7 mg/kg i.m., Bayern) and midazolam (0.6 mg/kg i.m., B. Braun). After sedation, the animal was bled (8 mL of blood was collected for downstream analysis) and weighed. The animal was euthanized with embutramide (0.1 mL/kg i.v., T61 MSD). Once death was confirmed by the veterinarian, the collection of the tissue samples was initiated. Samples from the brain regions, spinal cord, and liver were collected for further analysis.

DNA and RNA extraction from NHP tissues

DNA from NHP tissues was isolated using phenol:chloroform extraction after proteinase K digestion following previously published protocols.[34] RNA from NHP tissues was extracted using previously published TriReagent (Sigma, Cat# T9424):chloroform protocol.[34]

Reverse transcription of extracted RNA

Extracted RNA was incubated with TURBO DNase (Invitrogen, Cat# AM1907) for 3 h at 37°C, followed by incubation with DNAse inactivation reagent according to the manufacturer's instructions. The DNase-treated RNA was then used for cDNA synthesis using the SuperScript IV First-Strand Synthesis System (Invitrogen, Cat# 18091050) following the manufacturer's instructions and primed with Oligo(dT).

Biodistribution analysis

VCNs in NHP tissues were determined by ddPCR after tissue processing and extraction of total DNA. Vector genome content in each tissue was determined by digesting 60 ng genomic DNA per reaction with EcoRI-HF (NEB, Cat# R3101S) and quantified by ddPCR using eGFP primers (eGFP_F: 5′-TCAAGATCCGCCACAACATC-3′; eGFP_R: 5′-TTCTCGTTGGGGTCTTTGCT-3′) and NHP actin primers (NHP-β-actin_F: 5′-CAACGAGCGGTTCCGCTG-3′, NHP-β-actin_R: 5′-CAGCACTGTGTTGGCGTACAG-3′) as a reference.

Barcode amplification, NGS, and distribution analysis

The NGS amplicons were prepared and analyzed as previously published.[34] Briefly, 100 ng of extracted total DNA or 3 μL cDNA product was used to PCR amplify the AAV BC region using one of six available forward primers (BC_F_1–6; barcoded to allow multiplexing of different samples) and a universal reverse primer (BC_R: 5′-GCTGGCAACTAGAAGGCACAG-3′). PCR was performed using the Q5 high-fidelity DNA polymerase (NEB, Cat# M0491L). NGS reads from the DNA and cDNA populations were normalized to the reads from the pre-injection mix and displayed as a percentage of total reads.

Statistical analysis

All data were analyzed using Microsoft Excel and GraphPad Prism. Statistical significance was assessed using GraphPad Prism 10 software. Statistically significant differences in the distribution and intensity of eGFP expression *in vivo* were detected using one-way ANOVA with Tukey's multiple comparisons test. A p value of less than 0.05 was considered statistically significant.

DATA AND CODE AVAILABILITY

All of the data generated or analyzed during this study are available from the corresponding authors upon request.

SUPPLEMENTAL INFORMATION

Supplemental information can be found online at https://doi.org/10.1016/j.omtn.2024.102264.

ACKNOWLEDGMENTS

This research was supported by the NSW Health Early-Mid Career Gene and Cell Therapy Grants Program (grant K5126/RD665 to M.D.). The work of L.L. was supported by project grants from the Australian National Health and Medical Research Council (NHMRC) (APP2021305, APP2019968, and APP1161583) and a

research grant from the National Science Centre, Republic of Poland (OPUS-21) (2021/41/B/NZ5/01671). This research was also supported by the National Health and Medical Research Council, Medical Research Future Fund (MRF 2008912) and the NSW Luminesce Alliance (PPM1 K5116/RD274). Luminesce Alliance was established with the support of the Government of New South Wales to coordinate and integrate pediatric research in New South Wales. A.G.C, is the AI and Val Rosenstrauss Fellow, supported by the Rebecca Cooper Foundation. The authors would like to thank the Imaging Facility of CMRI. Figure 6A was generated with BioRender (https://biorender.com).

AUTHOR CONTRIBUTIONS

Conceptualization: M.D., A.G.-C., G.G-A., and L.L.; funding acquisition: M.D., C.U., G.G.-A., P.G.R.B.; S.M., A.G.-C., and L.L; investigation: M.D., T.N., J.M., D.N., A.W., S.L. J.L.L., A.F.V., and R.B.; methodology: M.D., T.N., C.U., G.G-A., P.G.R.B., S.M., and A.G.-C.; Software: S.S.; supervision: M.D., J.M., and L.L.; visualization: M.D. and J.M.; writing – original draft: M.D., J.M., S.M., and L.L.; all authors reviewed, edited, and commented on the manuscript.

DECLARATION OF INTERESTS

M.D., J.M., A.W., and L.L. are inventors on patent applications filed by Children's Medical Research Institute related to AAV capsid sequences, *in vivo* function of novel AAV variants and AAV selection platforms. L.L. is co-founder and owns equity in Exigen Biotherapeutics. L.L. and A.W. have consulted on technologies addressed in this paper. L.L. has stock and/or equity in companies with technology broadly related to this paper.

REFERENCES

1. Buning, H., and Srivastava, A. (2019). Capsid Modifications for Targeting and Improving the Efficacy of AAV Vectors. Mol. Ther. Methods Clin. Dev. *12*, 248–265. https://doi.org/10.1016/j.omtm.2019.01.008.
2. Becker, J., Fakhiri, J., and Grimm, D. (2022). Fantastic AAV Gene Therapy Vectors and How to Find Them-Random Diversification, Rational Design and Machine Learning. Pathogens *11*, 756. https://doi.org/10.3390/pathogens11070756.
3. Pupo, A., Fernández, A., Low, S.H., François, A., Suárez-Amarán, L., and Samulski, R.J. (2022). AAV vectors: The Rubik's cube of human gene therapy. Mol. Ther. *30*, 3515–3541. https://doi.org/10.1016/j.ymthe.2022.09.015.
4. Mullard, A. (2023). FDA approves first haemophilia B gene therapy. Nat. Rev. Drug Discov. *22*, 7. https://doi.org/10.1038/d41573-022-00199-8.
5. Mullard, A. (2023). FDA approves first gene therapy for Duchenne muscular dystrophy, despite internal objections. Nat. Rev. Drug Discov. *22*, 610. https://doi.org/10.1038/d41573-023-00103-y.
6. Kuzmin, D.A., Shutova, M.V., Johnston, N.R., Smith, O.P., Fedorin, V.V., Kukushkin, Y.S., van der Loo, J.C.M., and Johnstone, E.C. (2021). The clinical landscape for AAV gene therapies. Nat. Rev. Drug Discov. *20*, 173–174. https://doi.org/10.1038/d41573-021-00017-7.
7. Au, H.K.E., Isalan, M., and Mielcarek, M. (2021). Gene Therapy Advances: A Meta-Analysis of AAV Usage in Clinical Settings. Front. Med. *8*, 809118. https://doi.org/10.3389/fmed.2021.809118.
8. Deverman, B.E., Ravina, B.M., Bankiewicz, K.S., Paul, S.M., and Sah, D.W.Y. (2018). Gene therapy for neurological disorders: progress and prospects. Nat. Rev. Drug Discov. *17*, 641–659. https://doi.org/10.1038/nrd.2018.110.
9. Ojala, D.S., Amara, D.P., and Schaffer, D.V. (2015). Adeno-associated virus vectors and neurological gene therapy. Neuroscientist *21*, 84–98. https://doi.org/10.1177/1073858414521870.
10. Edelmann, M.J., and Maegawa, G.H.B. (2020). CNS-Targeting Therapies for Lysosomal Storage Diseases: Current Advances and Challenges. Front. Mol. Biosci. *7*, 559804. https://doi.org/10.3389/fmolb.2020.559804.
11. Xu, X., Chen, W., Zhu, W., Chen, J., Ma, B., Ding, J., Wang, Z., Li, Y., Wang, Y., and Zhang, X. (2021). Adeno-associated virus (AAV)-based gene therapy for glioblastoma. Cancer Cell Int. *21*, 76. https://doi.org/10.1186/s12935-021-01776-4.
12. Chand, D., Mohr, F., McMillan, H., Tukov, F.F., Montgomery, K., Kleyn, A., Sun, R., Tauscher-Wisniewski, S., Kaufmann, P., and Kullak-Ublick, G. (2021). Hepatotoxicity following administration of onasemnogene abeparvovec (AVXS-101) for the treatment of spinal muscular atrophy. J. Hepatol. *74*, 560–566. https://doi.org/10.1016/j.jhep.2020.11.001.
13. Mullard, A. (2021). Gene therapy community grapples with toxicity issues, as pipeline matures. Nat. Rev. Drug Discov. *20*, 804–805. https://doi.org/10.1038/d41573-021-00164-x.
14. Hinderer, C., Katz, N., Buza, E.L., Dyer, C., Goode, T., Bell, P., Richman, L.K., and Wilson, J.M. (2018). Severe Toxicity in Nonhuman Primates and Piglets Following High-Dose Intravenous Administration of an Adeno-Associated Virus Vector Expressing Human SMN. Hum. Gene Ther. *29*, 285–298. https://doi.org/10.1089/hum.2018.015.
15. Ronzitti, G., Gross, D.A., and Mingozzi, F. (2020). Human Immune Responses to Adeno-Associated Virus (AAV) Vectors. Front. Immunol. *11*, 670. https://doi.org/10.3389/fimmu.2020.00670.
16. Louis Jeune, V., Joergensen, J.A., Hajjar, R.J., and Weber, T. (2013). Pre-existing anti-adeno-associated virus antibodies as a challenge in AAV gene therapy. Hum. Gene Ther. Methods *24*, 59–67. https://doi.org/10.1089/hgtb.2012.243.
17. Kadry, H., Noorani, B., and Cucullo, L. (2020). A blood-brain barrier overview on structure, function, impairment, and biomarkers of integrity. Fluids Barriers CNS *17*, 69. https://doi.org/10.1186/s12987-020-00230-3.
18. Chen, X., Lim, D.A., Lawlor, M.W., Dimmock, D., Vite, C.H., Lester, T., Tavakkoli, F., Sadhu, C., Prasad, S., and Gray, S.J. (2023). Biodistribution of Adeno-Associated Virus Gene Therapy Following Cerebrospinal Fluid-Directed Administration. Hum. Gene Ther. *34*, 94–111. https://doi.org/10.1089/hum.2022.163.
19. Bedbrook, C.N., Deverman, B.E., and Gradinaru, V. (2018). Viral Strategies for Targeting the Central and Peripheral Nervous Systems. Annu. Rev. Neurosci. *41*, 323–348. https://doi.org/10.1146/annurev-neuro-080317-062048.
20. Kondratov, O., Kondratova, L., Mandel, R.J., Coleman, K., Savage, M.A., Gray-Edwards, H.L., Ness, T.J., Rodriguez-Lebron, E., Bell, R.D., Rabinowitz, J., et al. (2021). A comprehensive study of a 29-capsid AAV library in a non-human primate central nervous system. Mol. Ther. *29*, 2806–2820. https://doi.org/10.1016/j.ymthe.2021.07.010.
21. Hudry, E., and Vandenberghe, L.H. (2019). Therapeutic AAV Gene Transfer to the Nervous System: A Clinical Reality. Neuron *101*, 839–862. https://doi.org/10.1016/j.neuron.2019.02.017.
22. Srivastava, A. (2016). In vivo tissue-tropism of adeno-associated viral vectors. Curr. Opin. Virol. *21*, 75–80. https://doi.org/10.1016/j.coviro.2016.08.003.
23. Li, C., and Samulski, R.J. (2020). Engineering adeno-associated virus vectors for gene therapy. Nat. Rev. Genet. *21*, 255–272. https://doi.org/10.1038/s41576-019-0205-4.
24. Hordeaux, J., Wang, Q., Katz, N., Buza, E.L., Bell, P., and Wilson, J.M. (2018). The Neurotropic Properties of AAV-PHP.B Are Limited to C57BL/6J Mice. Mol. Ther. *26*, 664–668. https://doi.org/10.1016/j.ymthe.2018.01.018.
25. Matsuzaki, Y., Konno, A., Mochizuki, R., Shinohara, Y., Nitta, K., Okada, Y., and Hirai, H. (2018). Intravenous administration of the adeno-associated virus-PHP.B capsid fails to upregulate transduction efficiency in the marmoset brain. Neurosci. Lett. *665*, 182–188. https://doi.org/10.1016/j.neulet.2017.11.049.
26. Lancaster, M.A., Renner, M., Martin, C.A., Wenzel, D., Bicknell, L.S., Hurles, M.E., Homfray, T., Penninger, J.M., Jackson, A.P., and Knoblich, J.A. (2013). Cerebral organoids model human brain development and microcephaly. Nature *501*, 373–379. https://doi.org/10.1038/nature12517.
27. Pasca, S.P. (2018). The rise of three-dimensional human brain cultures. Nature *553*, 437–445. https://doi.org/10.1038/nature25032.
28. Stanton, M.M., Hariani, H.N., Sorokin, J., Taylor, P.M., Modan, S., Rash, B.G., Rao, S.B., Enriquez, L., Quang, D., Hsu, P.K., et al. (2023). Cell specificity of

adeno-associated virus (AAV) serotypes in human cortical organoids. Preprint at bioRxiv. https://doi.org/10.1101/2023.04.13.536491.

29. Depla, J.A., Sogorb-Gonzalez, M., Mulder, L.A., Heine, V.M., Konstantinova, P., van Deventer, S.J., Wolthers, K.C., Pajkrt, D., Sridhar, A., and Evers, M.M. (2020). Cerebral Organoids: A Human Model for AAV Capsid Selection and Therapeutic Transgene Efficacy in the Brain. Mol. Ther. Methods Clin. Dev. *18*, 167–175. https://doi.org/10.1016/j.omtm.2020.05.028.

30. Cho, A.N., Bright, F., Morey, N., Au, C., Ittner, L.M., and Ke, Y.D. (2022). Efficient Gene Expression in Human Stem Cell Derived-Cortical Organoids Using Adeno Associated Virus. Cells *11*, 3194. https://doi.org/10.3390/cells11203194.

31. Fernando, M., Lee, S., Wark, J.R., Xiao, D., Lim, B.Y., O'Hara-Wright, M., Kim, H.J., Smith, G.C., Wong, T., Teber, E.T., et al. (2022). Differentiation of brain and retinal organoids from confluent cultures of pluripotent stem cells connected by nerve-like axonal projections of optic origin. Stem Cell Rep. *17*, 1476–1492. https://doi.org/10.1016/j.stemcr.2022.04.003.

32. Cabanes-Creus, M., Navarro, R.G., Zhu, E., Baltazar, G., Liao, S.H.Y., Drouyer, M., Amaya, A.K., Scott, S., Nguyen, L.H., Westhaus, A., et al. (2022). Novel human liver-tropic AAV variants define transferable domains that markedly enhance the human tropism of AAV7 and AAV8. Mol. Ther. Methods Clin. Dev. *24*, 88–101. https://doi.org/10.1016/j.omtm.2021.11.011.

33. Westhaus, A., Eamegdool, S.S., Fernando, M., Fuller-Carter, P., Brunet, A.A., Miller, A.L., Rashwan, R., Knight, M., Daniszewski, M., Lidgerwood, G.E., et al. (2023). AAV capsid bioengineering in primary human retina models. Sci. Rep. *13*, 21946. https://doi.org/10.1038/s41598-023-49112-2.

34. Westhaus, A., Cabanes-Creus, M., Rybicki, A., Baltazar, G., Navarro, R.G., Zhu, E., Drouyer, M., Knight, M., Albu, R.F., Ng, B.H., et al. (2020). High-Throughput In Vitro, Ex Vivo, and In Vivo Screen of Adeno-Associated Virus Vectors Based on Physical and Functional Transduction. Hum. Gene Ther. *31*, 575–589. https://doi.org/10.1089/hum.2019.264.

35. Chan, K.Y., Jang, M.J., Yoo, B.B., Greenbaum, A., Ravi, N., Wu, W.L., Sánchez-Guardado, L., Lois, C., Mazmanian, S.K., Deverman, B.E., and Gradinaru, V. (2017). Engineered AAVs for efficient noninvasive gene delivery to the central and peripheral nervous systems. Nat. Neurosci. *20*, 1172–1179. https://doi.org/10.1038/nn.4593.

36. Foust, K.D., Nurre, E., Montgomery, C.L., Hernandez, A., Chan, C.M., and Kaspar, B.K. (2009). Intravascular AAV9 preferentially targets neonatal neurons and adult astrocytes. Nat. Biotechnol. *27*, 59–65. https://doi.org/10.1038/nbt.1515.

37. Tanguy, Y., Biferi, M.G., Besse, A., Astord, S., Cohen-Tannoudji, M., Marais, T., and Barkats, M. (2015). Systemic AAVrh10 provides higher transgene expression than AAV9 in the brain and the spinal cord of neonatal mice. Front. Mol. Neurosci. *8*, 36. https://doi.org/10.3389/fnmol.2015.00036.

38. Duong, T.T., Lim, J., Vasireddy, V., Papp, T., Nguyen, H., Leo, L., Pan, J., Zhou, S., Chen, H.I., Bennett, J., and Mills, J.A. (2019). Comparative AAV-eGFP Transgene Expression Using Vector Serotypes 1-9, 7m8, and 8b in Human Pluripotent Stem Cells, RPEs, and Human and Rat Cortical Neurons. Stem Cells Int. *2019*, 7281912. https://doi.org/10.1155/2019/7281912.

39. Paulk, N.K., Pekrun, K., Zhu, E., Nygaard, S., Li, B., Xu, J., Chu, K., Leborgne, C., Dane, A.P., Haft, A., et al. (2018). Bioengineered AAV Capsids with Combined High Human Liver Transduction *In Vivo* and Unique Humoral Seroreactivity. Mol. Ther. *26*, 289–303. https://doi.org/10.1016/j.ymthe.2017.09.021.

40. Dalkara, D., Byrne, L.C., Klimczak, R.R., Visel, M., Yin, L., Merigan, W.H., Flannery, J.G., and Schaffer, D.V. (2013). *In vivo*-directed evolution of a new adeno-associated virus for therapeutic outer retinal gene delivery from the vitreous. Sci. Transl. Med. *5*, 189ra76. https://doi.org/10.1126/scitranslmed.3005708.

41. Gonzalez-Sandoval, A., Pekrun, K., Tsuji, S., Zhang, F., Hung, K.L., Chang, H.Y., and Kay, M.A. (2023). The AAV capsid can influence the epigenetic marking of rAAV delivered episomal genomes in a species dependent manner. Nat. Commun. *14*, 2448. https://doi.org/10.1038/s41467-023-38106-3.

42. Tordo, J., O'Leary, C., Antunes, A.S.L.M., Palomar, N., Aldrin-Kirk, P., Basche, M., Bennett, A., D'Souza, Z., Gleitz, H., Godwin, A., et al. (2018). A novel adeno-associated virus capsid with enhanced neurotropism corrects a lysosomal transmembrane enzyme deficiency. Brain *141*, 2014–2031. https://doi.org/10.1093/brain/awy126.

43. Sullivan, J.A., Stanek, L.M., Lukason, M.J., Bu, J., Osmond, S.R., Barry, E.A., O'Riordan, C.R., Shihabuddin, L.S., Cheng, S.H., and Scaria, A. (2018). Rationally designed AAV2 and AAVrh8R capsids provide improved transduction in the retina and brain. Gene Ther. *25*, 205–219. https://doi.org/10.1038/s41434-018-0017-8.

44. Meyer, K., Ferraiuolo, L., Schmelzer, L., Braun, L., McGovern, V., Likhite, S., Michels, O., Govoni, A., Fitzgerald, J., Morales, P., et al. (2015). Improving single injection CSF delivery of AAV9-mediated gene therapy for SMA: a dose-response study in mice and nonhuman primates. Mol. Ther. *23*, 477–487. https://doi.org/10.1038/mt.2014.210.

45. Bey, K., Deniaud, J., Dubreil, L., Joussemet, B., Cristini, J., Ciron, C., Hordeaux, J., Le Boulc'h, M., Marche, K., Maquigneau, M., et al. (2020). Intra-CSF AAV9 and AAVrh10 Administration in Nonhuman Primates: Promising Routes and Vectors for Which Neurological Diseases? Mol. Ther. Methods Clin. Dev. *17*, 771–784. https://doi.org/10.1016/j.omtm.2020.04.001.

46. Ertl, H.C.J. (2022). Immunogenicity and toxicity of AAV gene therapy. Front. Immunol. *13*, 975803. https://doi.org/10.3389/fimmu.2022.975803.

47. Ramamurthy, R.M., Atala, A., Porada, C.D., and Almeida-Porada, G. (2022). Organoids and microphysiological systems: Promising models for accelerating AAV gene therapy studies. Front. Immunol. *13*, 1011143. https://doi.org/10.3389/fimmu.2022.1011143.

48. Keng, C.T., Guo, K., Liu, Y.C., Shen, K.Y., Lim, D.S., Lovatt, M., Ang, H.P., Mehta, J.S., and Chew, W.L. (2022). Multiplex viral tropism assay in complex cell populations with single-cell resolution. Gene Ther. *29*, 555–565. https://doi.org/10.1038/s41434-022-00360-3.

49. Bruinstroop, E., Cano, G., Vanderhorst, V.G.J.M., Cavalcante, J.C., Wirth, J., Sena-Esteves, M., and Saper, C.B. (2012). Spinal projections of the A5, A6 (locus coeruleus), and A7 noradrenergic cell groups in rats. J. Comp. Neurol. *520*, 1985–2001. https://doi.org/10.1002/cne.23024.

50. Gebhart, G.F. (2004). Descending modulation of pain. Neurosci. Biobehav. Rev. *27*, 729–737. https://doi.org/10.1016/j.neubiorev.2003.11.008.

51. Fink, K.L., Strittmatter, S.M., and Cafferty, W.B.J. (2015). Comprehensive Corticospinal Labeling with mu-crystallin Transgene Reveals Axon Regeneration after Spinal Cord Trauma in ngr1-/- Mice. J. Neurosci. *35*, 15403–15418. https://doi.org/10.1523/JNEUROSCI.3165-15.2015.

52. Chen, T., Taniguchi, W., Chen, Q.Y., Tozaki-Saitoh, H., Song, Q., Liu, R.H., Koga, K., Matsuda, T., Kaito-Sugimura, Y., Wang, J., et al. (2018). Top-down descending facilitation of spinal sensory excitatory transmission from the anterior cingulate cortex. Nat. Commun. *9*, 1886. https://doi.org/10.1038/s41467-018-04309-2.

53. Cabanes-Creus, M., Navarro, R.G., Liao, S.H.Y., Scott, S., Carlessi, R., Roca-Pinilla, R., Knight, M., Baltazar, G., Zhu, E., Jones, M., et al. (2023). Characterization of the humanized FRG mouse model and development of an AAV-LK03 variant with improved liver lobular biodistribution. Mol. Ther. Methods Clin. Dev. *28*, 220–237. https://doi.org/10.1016/j.omtm.2022.12.014.

54. Cabanes-Creus, M., Hallwirth, C.V., Westhaus, A., Ng, B.H., Liao, S.H.Y., Zhu, E., Navarro, R.G., Baltazar, G., Drouyer, M., Scott, S., et al. (2020). Restoring the natural tropism of AAV2 vectors for human liver. Sci. Transl. Med. *12*, eaba3312. https://doi.org/10.1126/scitranslmed.aba3312.

55. Cabanes-Creus, M., Westhaus, A., Navarro, R.G., Baltazar, G., Zhu, E., Amaya, A.K., Liao, S.H.Y., Scott, S., Sallard, E., Dilworth, K.L., et al. (2020). Attenuation of Heparan Sulfate Proteoglycan Binding Enhances In Vivo Transduction of Human Primary Hepatocytes with AAV2. Mol. Ther. Methods Clin. Dev. *17*, 1139–1154. https://doi.org/10.1016/j.omtm.2020.05.004.

56. Cabanes Creus, M. (2019). Novel AAV engineering technology: identification of improved AAV variants for gene addition and genome engineering in primary human cells. https://discovery.ucl.ac.uk/id/eprint/10071599/.

57. Schmahmann, J.D. (2004). Disorders of the cerebellum: ataxia, dysmetria of thought, and the cerebellar cognitive affective syndrome. J. Neuropsychiatry Clin. Neurosci. *16*, 367–378. https://doi.org/10.1176/jnp.16.3.367.

58. Lancaster, M.A., and Knoblich, J.A. (2014). Generation of cerebral organoids from human pluripotent stem cells. Nat. Protoc. *9*, 2329–2340. https://doi.org/10.1038/nprot.2014.158.

59. Khan, I.F., Hirata, R.K., and Russell, D.W. (2011). AAV-mediated gene targeting methods for human cells. Nat. Protoc. *6*, 482–501. https://doi.org/10.1038/nprot.2011.301.

60. Strobel, B., Miller, F.D., Rist, W., and Lamla, T. (2015). Comparative Analysis of Cesium Chloride- and Iodixanol-Based Purification of Recombinant Adeno-Associated Viral Vectors for Preclinical Applications. Hum. Gene Ther. Methods *26*, 147–157. https://doi.org/10.1089/hgtb.2015.051.

61. Schindelin, J., Arganda-Carreras, I., Frise, E., Kaynig, V., Longair, M., Pietzsch, T., Preibisch, S., Rueden, C., Saalfeld, S., Schmid, B., et al. (2012). Fiji: an open-source platform for biological-image analysis. Nat. Methods *9*, 676–682. https://doi.org/10.1038/nmeth.2019.

62. Salas, D., Kwikkers, K.L., Zabaleta, N., Bazo, A., Petry, H., van Deventer, S.J., Aseguinolaza, G.G., and Ferreira, V. (2019). Immunoadsorption enables successful rAAV5-mediated repeated hepatic gene delivery in nonhuman primates. Blood Adv. *3*, 2632–2641. https://doi.org/10.1182/bloodadvances.2019000380.

OMTN, Volume *35*

Supplemental information

Enhanced AAV transduction across preclinical CNS models: A comparative study in human brain organoids with cross-species evaluations

Matthieu Drouyer, Jessica Merjane, Teodora Nedelkoska, Adrian Westhaus, Suzanne Scott, Scott Lee, Peter G.R. Burke, Simon McMullan, Jose L. Lanciego, Ana F. Vicente, Ricardo Bugallo, Carmen Unzu, Gloria González-Aseguinolaza, Anai Gonzalez-Cordero, and Leszek Lisowski

Supplemental Figures

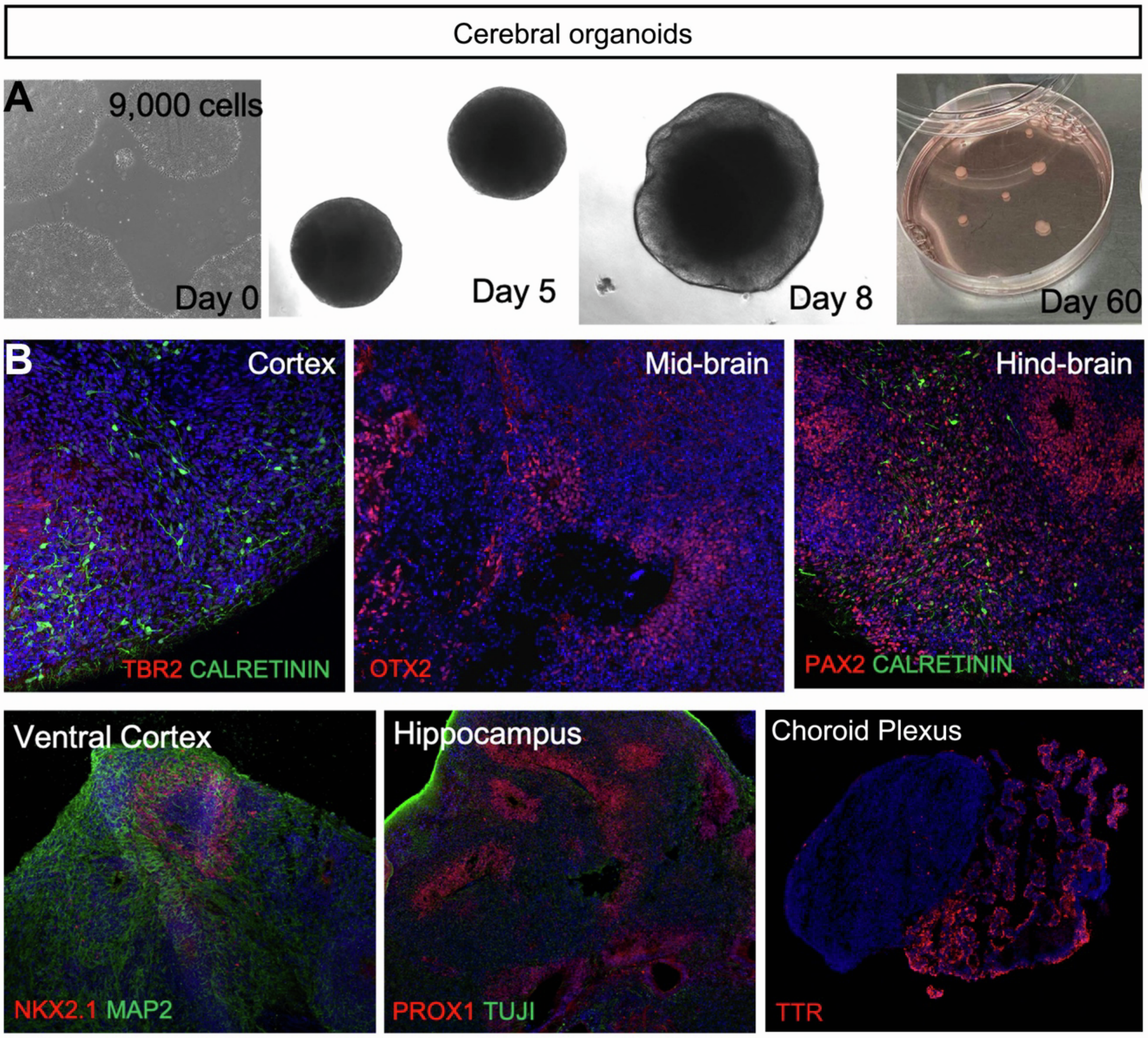

Figure S1. Characterization of human cerebral organoids. (**A**) Bright-field images of organoids induced from human iPSCs. (**B**) Cerebral organoids recapitulate various brain region identities. Staining for various brain region identities: cortex, Tbr2; mid-brain, Otx2; hind-brain, Pax2, ventral cortex, Nkx2.1; hippocampus, Prox1 and choroid plexus, TTR. Immunohistochemistry for neuronal cell marker (Tuj1, MAP2 and Calretinin). Nuclei were visualized with DAPI (blue).

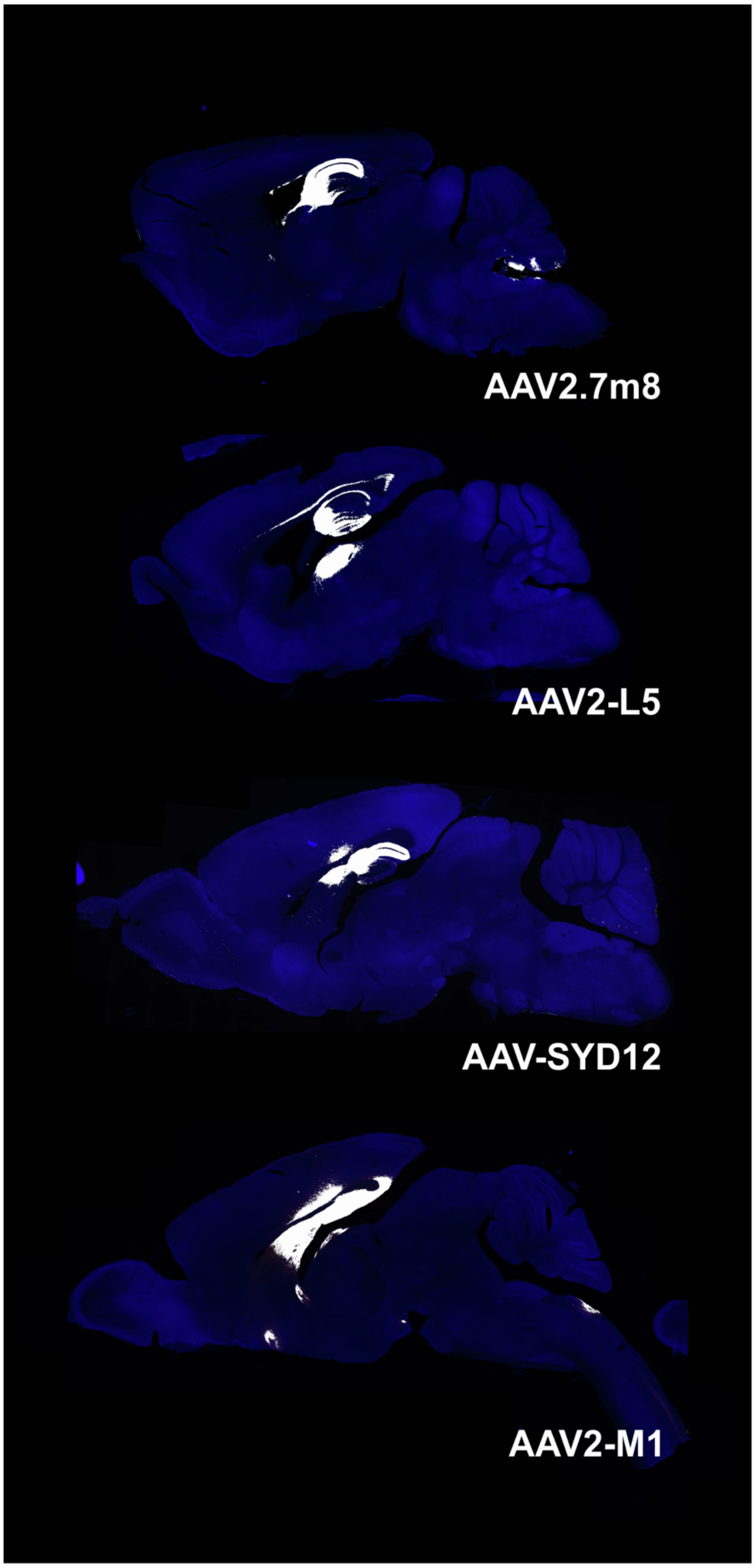

Figure S2. Threshold masks used for quantification of vector efficacy. An ImageJ macro was used to identify pixels that exceeded a threshold fluorescence intensity; the area of each image contained by the mask, and the average intensity of pixels within, were compared between vectors. Masks corresponding to images shown in Figure 5 are shown for comparison.

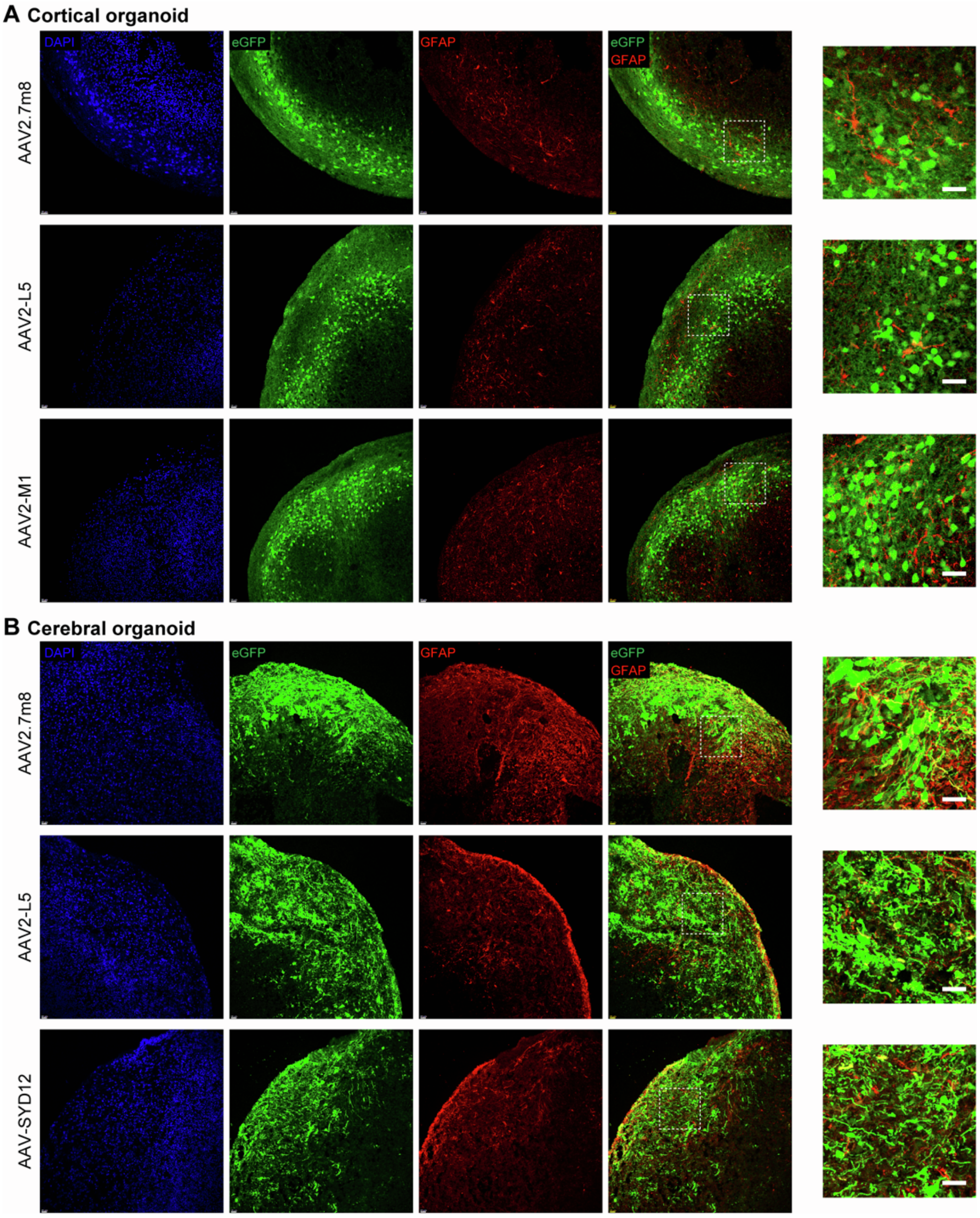

Figure S3. Immunofluorescence analysis of GFAP in cortical and cerebral organoids. (**A**) (left) eGFP reporter expression in cortical organoids following transduction with AAV2.7m8, AAV2-L5 and AAV2-M1 variants. Staining with GFAP was used to identify glia cells (n = 1 organoid, dose: 1×10^{10} vg per organoid, 2 weeks of expression). (right) High magnification images of selected region. Blue: DAPI (nuclei); green: vector-encoded eGFP; red: astrocytes and an overlay of green and red. Scale bars, 20µm. (**B**) (left) eGFP reporter expression in cerebral organoids following transduction with AAV2.7m8, AAV2-L5 and AAV-SYD12 variants. Staining with GFAP was used to identify glia cells (n = 1 organoid, dose: 1×10^{10} vg per organoid, 2 weeks of expression). (right) High magnification images of selected region. Blue: DAPI (nuclei); green: vector-encoded eGFP; red: astrocytes and an overlay of green and red. Scale bars, 20µm.

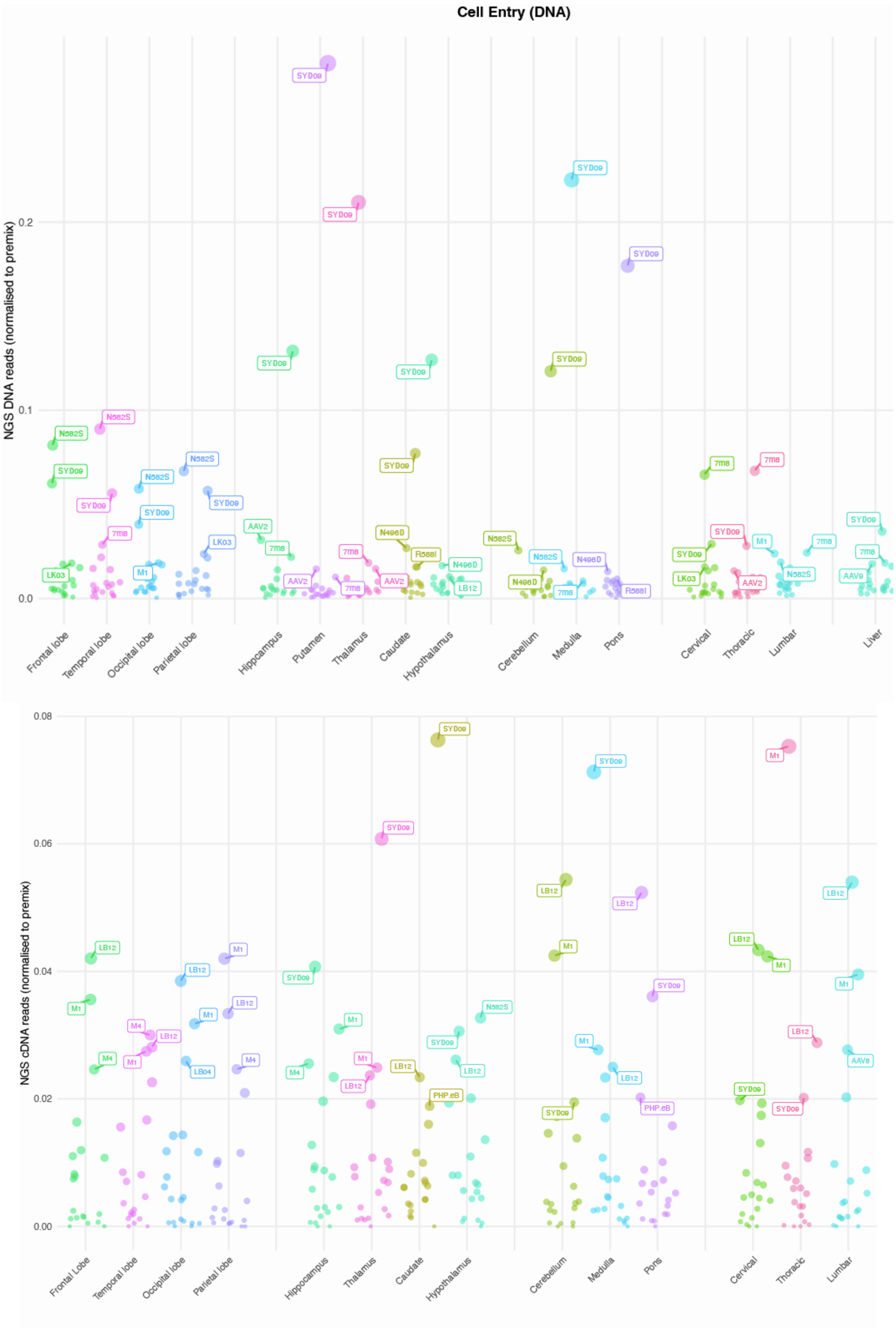

Figure S3. AAV biodistribution in CNS areas and liver in non-human primate following Intra-cisterna magna injection. NGS analysis of the transduction efficiency of each AAV variant at cell entry (DNA reads) and functional expression (RNA/cDNA reads) for each region within the CNS and liver. Bubble plots depict the percentage of NGS reads for each variant. Each bubble represents one AAV variant. Bubble size is proportional to percentage number. Top three AAV serotypes for each tissue is indicated.

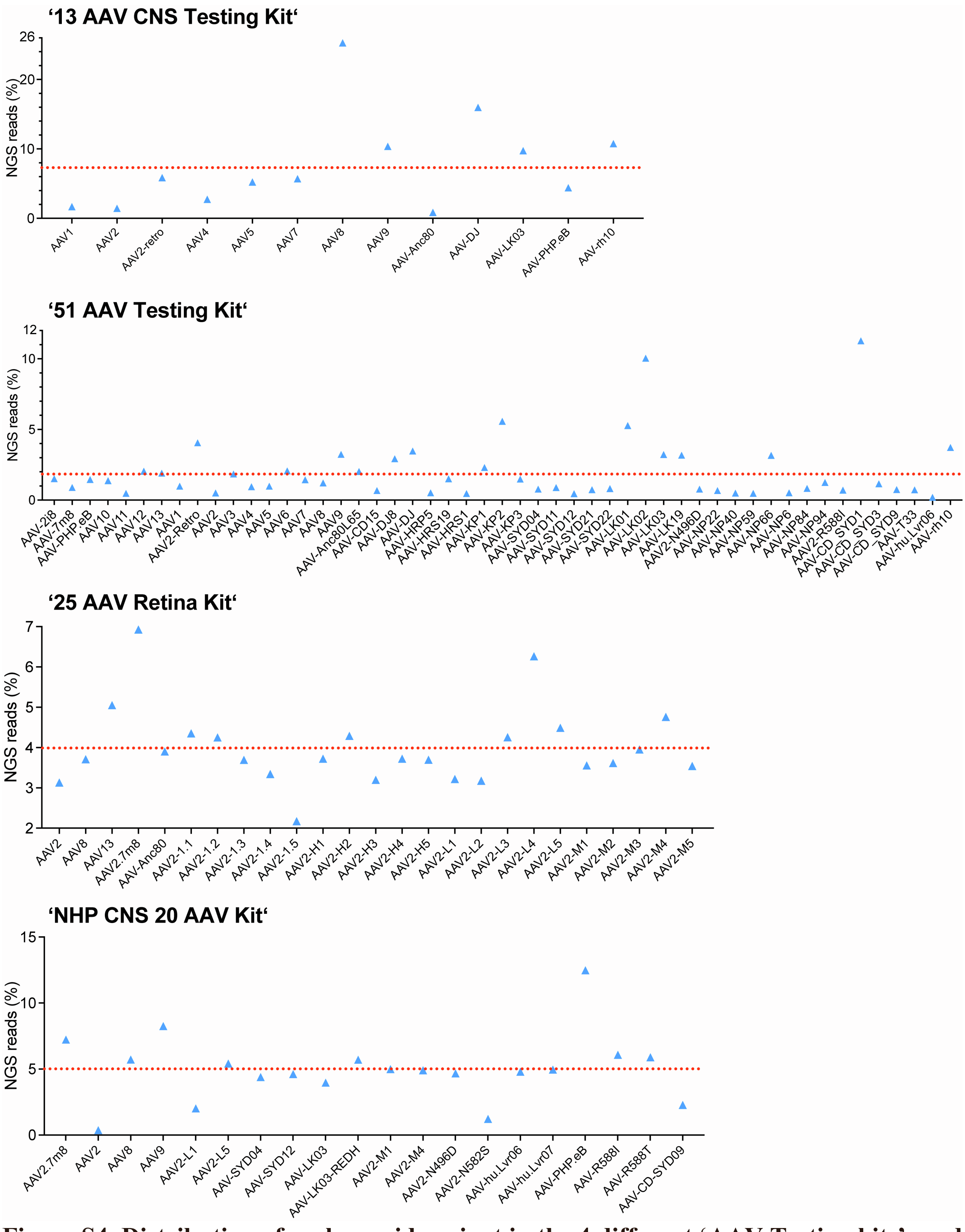

Figure S4. Distribution of each capsid variant in the 4 different ‘AAV Testing kits’ used in this study. The expected percentage contribution of individual variants is indicated by the red dotted line.

Table S1. AAV capsid variants used in the study.

AAV Variant	Capsid Origin	AAV CNS Testing	51 AAV Testing kit	25 AAV Retina kit	NHP CNS 20 AAV kit	Reference
AAV1	Natural serotype	X	X			PMID: 14325163
AAV2	Natural serotype	X	X	X	X	PMID: 5227666
AAV3b	Natural serotype		X			PMID: 5227666
AAV4	Natural serotype	X	X			PMID: 9261407
AAV5	Natural serotype	X	X			PMID: 6324476
AAV6	Natural serotype		X			PMID: 5227666
AAV7	Natural serotype	X	X			PMID: 12192090
AAV8	Natural serotype	X	X	X	X	PMID: 12192090
AAV9	Natural serotype	X	X		X	PMID: 15163731
AAV10	Natural serotype		X			PMID: 15567432
AAV11	Natural serotype		X			PMID: 15567432
AAV12	Natural serotype		X			PMID: 18045941
AAV13	Natural serotype		X	X		PMID: 18524816
AAV-rh10	Natural isolate	X	X			PMID: 12716974
AAV-DJ	DNA family shuffling	X	X			PMID: 18400866
AAV-DJ8	DNA family shuffling		X			PMID: 18400866
AAV-LK01	DNA family shuffling		X			PMID: 24390344
AAV-LK02	DNA family shuffling		X			PMID: 24390344
AAV-LK03	DNA family shuffling	X	X		X	PMID: 24390344
AAV-LK19	DNA family shuffling		X			PMID: 24390344
AAV-NP22	DNA family shuffling		X			PMID: 30101152
AAV-NP40	DNA family shuffling		X			PMID: 29055620
AAV-NP59	DNA family shuffling		X			PMID: 29055620
AAV-NP6	DNA family shuffling		X			PMID: 30101152
AAV-NP66	DNA family shuffling		X			PMID: 30101152
AAV-NP84	DNA family shuffling		X			PMID: 29055620
AAV-NP94	DNA family shuffling		X			PMID: 30101152
AAV-7m8	Peptide display on AAV2		X	X	X	PMID: 23761039
AAV-Anc80L65	Reconstructed capsid	X	X	X		PMID: 26235624
AAV-PHP.eB	Peptide display on AAV9	X	X		X	PMID: 28671695
AAV-2i8	Domain swapping		X			PMID: 20037580
AAV2-retro	Peptide display on AAV2	X	X			PMID: 27720486
AAV-CD15	DNA family shuffling		X			Our lab
AAV-HRP5	DNA family shuffling		X			Our lab
AAV-HRS19	DNA family shuffling		X			Our lab
AAV-HRS1	DNA family shuffling		X			Our lab
AAV-KP1	DNA family shuffling		X			PMID: 31723052
AAV-KP2	DNA family shuffling		X			PMID: 31723052
AAV-KP3	DNA family shuffling		X			PMID: 31723052
AAV-SYD04	DNA family shuffling		X		X	Our lab
AAV-SYD11	DNA family shuffling		X			Our lab
AAV-SYD12	DNA family shuffling		X		X	Our lab
AAV2-RC01	Peptide display on AAV2		X			Our lab
AAV2-RC02	Peptide display on AAV2		X			Our lab
AAV2-N496D	Rational Design		X		X	Our lab
AAV2-R588I	Rational Design		X		X	Our lab
AAV-CD-SYD01	DNA family shuffling		X			Our lab
AAV-CD-SYD03	DNA family shuffling		X			Our lab
AAV-CD-SYD09	DNA family shuffling		X		X	Our lab
AAV-T33	DNA family shuffling		X			Our lab
AAV-hu.Lvr06	Natural isolate		X		X	Our lab
AAV2-N582S	Rational Design				X	Our lab
AAV2-R588T	Rational Design				X	Our lab
AAV-LK03-REDH	Rational Design				X	Our lab
AAV-hu.Lvr07	Natural isolate				X	Our lab
AAV2-1.1	Peptide display on AAV2			X		Our lab
AAV2-1.2	Peptide display on AAV2			X		Our lab
AAV2-1.3	Peptide display on AAV2			X		Our lab
AAV2-1.4	Peptide display on AAV2			X		Our lab
AAV2-1.5	Peptide display on AAV2			X		Our lab
AAV2-H1	Peptide display on AAV2			X		Our lab
AAV2-H2	Peptide display on AAV2			X		Our lab
AAV2-H3	Peptide display on AAV2			X		Our lab
AAV2-H4	Peptide display on AAV2			X		Our lab
AAV2-H5	Peptide display on AAV2			X		Our lab
AAV2-L1	Peptide display on AAV2			X	X	Our lab
AAV2-L2	Peptide display on AAV2			X		Our lab
AAV2-L3	Peptide display on AAV2			X		Our lab
AAV2-L4	Peptide display on AAV2			X		Our lab
AAV2-L5	Peptide display on AAV2			X	X	Our lab
AAV2-M1	Peptide display on AAV2			X	X	Our lab
AAV2-M2	Peptide display on AAV2			X		Our lab
AAV2-M3	Peptide display on AAV2			X		Our lab
AAV2-M4	Peptide display on AAV2			X	X	Our lab
AAV2-M5	Peptide display on AAV2			X		Our lab
